# Efficient gene knockout in primary human and murine myeloid cells by non-viral delivery of CRISPR-Cas9

**DOI:** 10.1084/jem.20191692

**Published:** 2020-05-01

**Authors:** Emily C. Freund, Jaclyn Y. Lock, Jaehak Oh, Timurs Maculins, Lelia Delamarre, Christopher J. Bohlen, Benjamin Haley, Aditya Murthy

**Affiliations:** 1 Department of Molecular Biology, Genentech, South San Francisco, CA; 2 Department of Cancer Immunology, Genentech, South San Francisco, CA; 3 Department of Neuroscience, Genentech, South San Francisco, CA

## Abstract

Myeloid cells play critical and diverse roles in mammalian physiology, including tissue development and repair, innate defense against pathogens, and generation of adaptive immunity. As cells that show prolonged recruitment to sites of injury or pathology, myeloid cells represent therapeutic targets for a broad range of diseases. However, few approaches have been developed for gene editing of these cell types, likely owing to their sensitivity to foreign genetic material or virus-based manipulation. Here we describe optimized strategies for gene disruption in primary myeloid cells of human and murine origin. Using nucleofection-based delivery of Cas9-ribonuclear proteins (RNPs), we achieved near population-level genetic knockout of single and multiple targets in a range of cell types without selection or enrichment. Importantly, we show that cellular fitness and response to immunological stimuli is not significantly impacted by the gene editing process. This provides a significant advance in the study of myeloid cell biology, thus enabling pathway discovery and drug target validation across species in the field of innate immunity.

## Teaser

Highly efficient gene editing in primary myeloid cells is made possible by optimized non-viral delivery of CRISPR-Cas9. This enables single and multiplexed gene knockout in monocytes, macrophages, and dendritic cells to advance innate immunity research and cell therapy.

## Graphical Abstract

**Figure:**
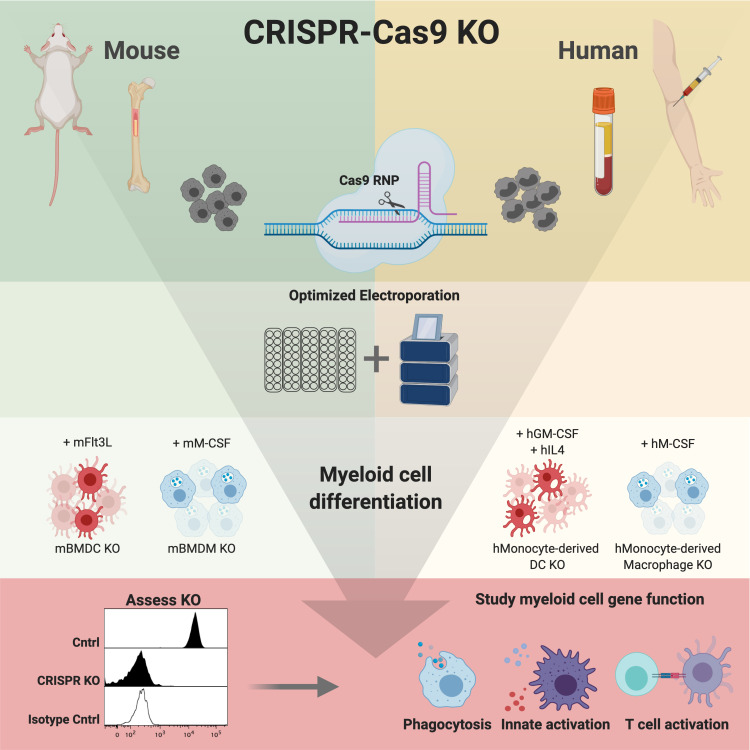

## Introduction

Myeloid cells constitute the innate immune system, providing a first line of defense against pathogens while also generating the requisite inflammation for optimal adaptive immunity (Bassler et al., 2019; Geissmann and Mass, 2015; Ginhoux and Guilliams, 2016; Jakubzick et al., 2017; Varol et al., 2015). Myeloid cell subsets include granulocytes, macrophages, monocytes, and dendritic cells (DCs). These cells are critical components of the tissue microenvironment, acting as effectors for direct killing of pathogens or infected cells, as phagocytes to clear dead cells or pathogens, as professional APCs to drive adaptive immunity, and finally, as modifiers of the microenvironment by generation of inflammatory or reparative factors such as cytokines. Strategies targeting myeloid cells have emerged as relevant for promoting antimicrobial or antitumor immunity, making research into the biology or modification of these cell types necessary for advancing immunomodulatory therapeutics (Baer et al., 2016; Broz et al., 2014; Edwards et al., 2019; Kuhn et al., 2013; Kumar et al., 2017; Perry et al., 2018; Stromnes et al., 2019). In spite of this, current research on innate immunity is largely restricted to transformed myeloid cell lines, virus-mediated gene delivery, myeloid cells derived from Cas9 knock-in mouse models, or other engineered murine genetic models (e.g., KO, inducible cell deletion, reporter; Baker and Masters, 2018; Hammerschmidt et al., 2018; Napier et al., 2016; Platt et al., 2014; Roberts et al., 2019). The lack of tools specifically for primary human and mouse myeloid cell gene editing is likely due to the high sensitivity these cells exhibit for sensing and responding to exogenous nucleic acids or pathogen invasion (e.g., viral vector or nucleic acid delivery), and consequently variable efficiency of editing (Hornung et al., 2006; Leyva et al., 2011; Bobadilla et al., 2013; Coch et al., 2013).

Gene editing by CRISPR-Cas9 technology has dramatically accelerated pathway discovery and analysis. Originally described as a form of adaptive microbial immunity against bacteriophages, CRISPR-based platforms have been developed to enable targeted gene editing and regulation in mammalian cells by directing DNA endonucleases (e.g., Cas9) toward specific genomic loci via user-defined guide RNA (gRNA) sequences (Simeonov and Marson, 2019). A vital factor for maximizing CRISPR-Cas9 activity in a target cell type is effective delivery and expression of the Cas9-gRNA ribonucleoprotein (RNP) complex (Doench, 2018). While this can generally be accomplished in immortalized or primary cell types by stable, typically virus-based expression of Cas9 and/or the gRNA, efficient gene editing would thus require a selection process (i.e., antibiotic or fluorescent protein), a specialized facility and protocol for working with biosafety level 2 viral vectors, and potentially Cas9-transgenic animal models (Parnas et al., 2015; Platt et al., 2014). Additionally, stable expression of Cas9, a bacterial protein, may limit in vivo application of this approach owing to potential antigen-specific reaction against Cas9 itself.

Through a comprehensive assessment of intracellular delivery conditions and Cas9 protein or synthetic gRNA variants, as well as intracellular delivery conditions, we have developed optimized, non-viral CRISPR-Cas9–based protocols for gene disruption in primary myeloid cells of human and murine origin. Focusing on non-granulocytic cells (i.e., monocytes, macrophages, and DCs), we demonstrate that delivery of Cas9-RNP complexes routinely generates >90% KO of single or multiple target genes without the need for cell selection. This is achieved in both differentiated primary myeloid cell populations and freshly isolated cells, enabling rapid loss-of-function gene assessment across donors or cells derived from pre-defined genetic backgrounds without compromising normal cell functions.

## Results

### Efficient genome engineering in murine bone marrow (BM)–derived macrophages (BMDMs) and DCs

**Figure S1.**
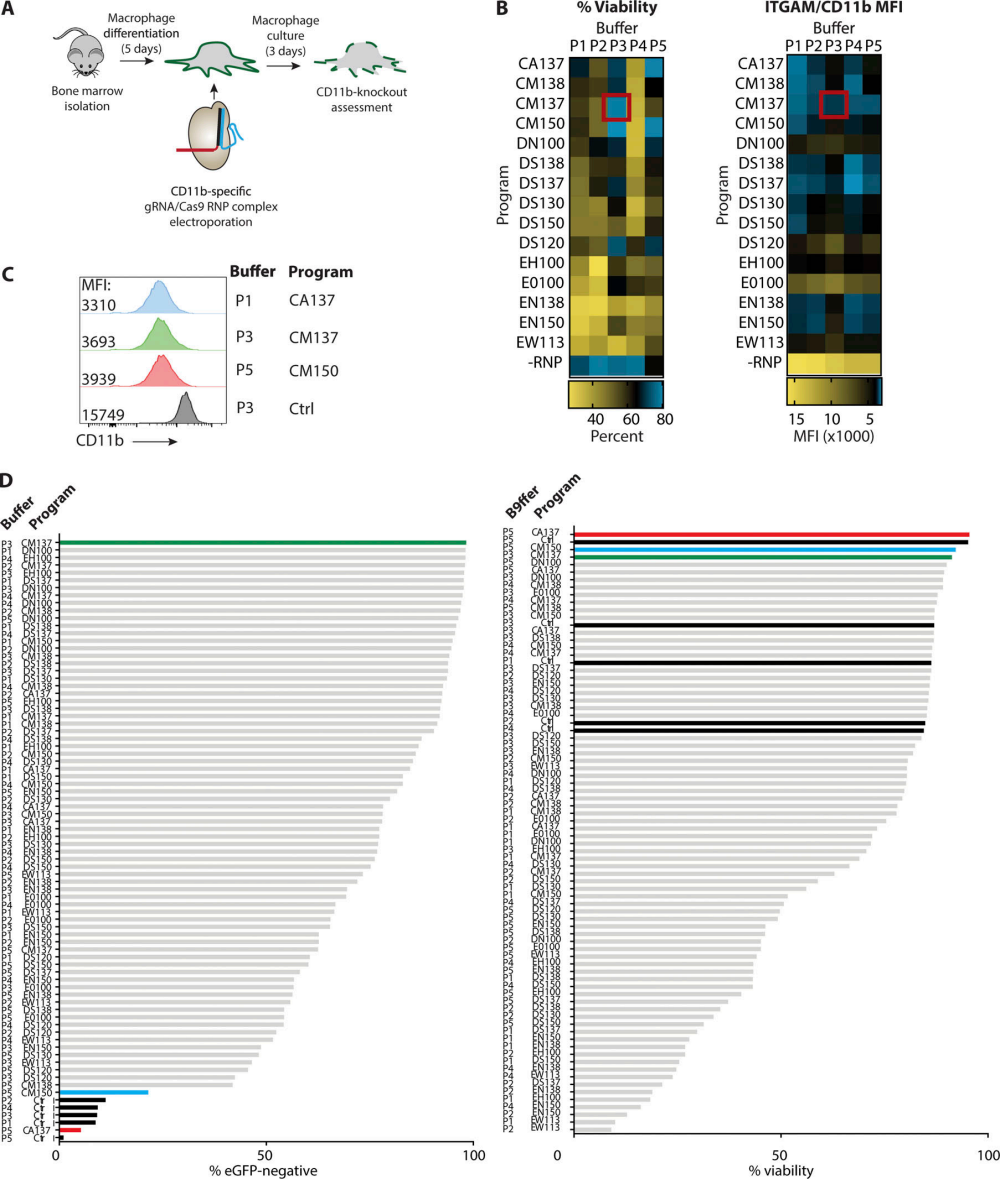
**Screening of optimal Cas9-RNP nucleofection protocols for KO in murine monocytes and BMDMs. (A)** Workflow for generation of murine BMDMs and screening of crRNA/Cas9-mediated CD11b KO. **(B)** Heatmaps depicting relative impact of nucleofection conditions on cell viability and CD11b MFI. Red box indicates the best condition (Buffer P3, program CM-137). **(C)** Representative histograms depicting MFI of CD11b for indicated nucleofection conditions following 5 d of culture in BMDM medium. **(D)** Ranking of nucleofection conditions for crRNA/Cas9-mediated eGFP KO and viability in monocyte-derived macrophages following 5 d of culture. Green bar: Buffer P3, Program CM-137. Blue bar: Buffer P5, Program CM-150. Red bar: Buffer P5, Program CA-137. Black bars: Nucleofection controls without crRNA-Cas9. All data are representative of one screening experiment.

Macrophages reside within diverse tissue niches in vivo, playing critical roles in regulating infection, inflammation, and tissue regeneration. Macrophages are derived from yolk-sac or hematopoietic progenitors during development or monocytes in later post-natal life (Geissmann et al., 2010; Varol et al., 2015). They are primary sensors of infection and tissue injury, with the ability to either promote inflammation and cellular and humoral immunity or drive tissue regeneration and potentially fibrosis (Wynn and Vannella, 2016). Murine BMDMs can be derived from hematopoietic progenitors by culture of total BM in the presence of CSF1/M-CSF, which generates large numbers of these cells for experimental use. BMDMs have proven invaluable for studying the function of macrophages and are widely used to understand innate immune signaling. We sought a non-viral, selection-free method of genetically modifying this cell type. We performed a flow cytometry–based screen in murine BMDMs to identify optimal Cas9-RNP electroporation (nucleofection)-based parameters for gene disruption, using the cell surface–localized protein CD11b (encoded from the *Itgam* locus, hereafter CD11b) as a model target (Fig. S1 A; gRNA sequences provided in Table S1).

Here, a matrix comprising five buffers and 15 nucleofection settings was used to define conditions that provide high efficiency of gene editing without an impact on cell viability. BMDMs were differentiated for 5 d in M-CSF, then used at 0.5 million cells per condition. Two distinct CRISPR RNAs (crRNAs) were designed to target the CD11b coding sequence and synthesized at Integrated DNA Technologies (IDT). Subsequently, these guides were annealed to transactivating crRNAs (tracrRNAs), complexed with *Streptococcus pyogenes* Cas9 (SpCas9, hereafter Cas9) protein (IDT Cas9 V2), and pooled to improve gene deletion probability. A 3:1 molar ratio of cr/tracrRNA:Cas9 was used as a starting condition (60 pmol or 10 µg Cas9 per reaction), as previously reported for lymphocytes (Seki and Rutz, 2018). After nucleofection, BMDMs were cultured in M-CSF for an additional 5 d. Viability and CD11b KO efficiency were compared by flow cytometry (Fig. S1, B and C). Although a majority of nucleofection conditions proved to be toxic (Fig. S1 B, % viability), we identified several conditions that preserved cell viability while inducing CD11b disruption (Fig. S1 B, CD11b mean fluorescence intensity [MFI]). Of these, the top three conditions revealed comparable loss of CD11b expression (Fig. S1 C), with buffer P3, program CM-137 maintaining the highest level of cell viability (red box, Fig. S1 B).

**Figure 1.**
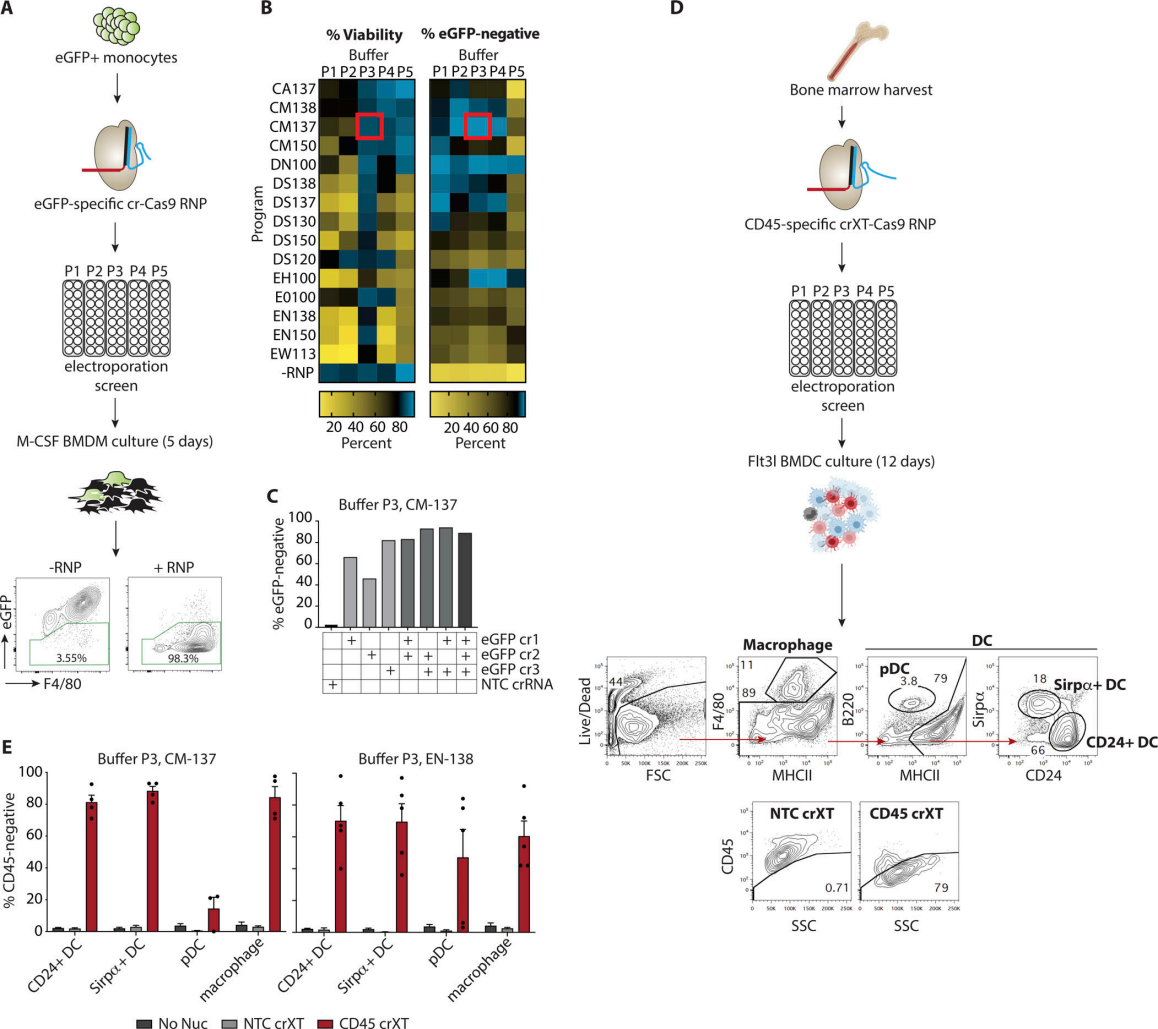
**Efficient gene editing in murine monocytes, macrophages, and DCs obtained from the BM. (A)** Workflow for screening crRNA-Cas9-RNP–mediated KO of eGFP in mouse monocytes. Representative flow cytometry plots show gating strategy for identifying eGFP KO, F4/80^+^ macrophages following 5 d of culture in M-CSF (the workflow was previously published in Lim et al., 2019). **(B)** Heatmaps depicting relative impact of nucleofection conditions on cell viability and eGFP KO. Red box indicates the best condition (Buffer P3, Program CM-137). **(C)** eGFP KO efficiency following nucleofection with single or pooled crRNAs. NTC, non-targeting control crRNA-Cas9-RNP. **(D)** Workflow for CD45 crRNAXT-Cas9-RNP–mediated KO in mouse BMDC cultures. Top flow-cytometry plots show gating strategy for identifying macrophage, pDC, CD24^+^ DC, and Sirpα^+^ DC cells. Bottom flow-cytometry plots depict representative gating strategy using NTC crRNAXT-Cas9-RNP as a control for determining CD45-negative cells in each cell population. **(E)** CD45 KO efficiency as measured by flow cytometry (experimental workflow and gating strategy shown in D) in BM-derived CD24^+^, Sirpα^+^, pDC, and macrophage cells nucleofected with Cas9-RNPs (IDT V3) loaded with NTC or CD45 targeting crRNAXT using either P3/CM-137 or P3/EN138 combinations. Data in B–D are from single screening experiments. Data in E are presented as mean ± SEM (*n* = 4–5) and collected from two independent experiments.

We next performed a similar study using monocytes isolated from femoral BM of eGFP-transgenic mice (expressing *egfp* under control of the *bactin* promoter, hereafter eGFP), this time using distinct crRNA sequences targeting eGFP (Chen et al., 2017) to assess whether population-level gene deletion can be obtained from freshly isolated and edited cells (Fig. 1 A). The ability to screen for loss of an intracellular marker such as eGFP precluded receptor internalization as a potential confounder in the previous assay. After Cas9-RNP nucleofection, monocytes were cultured in M-CSF for 5 d to generate monocyte-derived macrophages. Consistent with our findings for CD11b disruption, buffer P3, program CM-137 again produced the greatest loss of target (eGFP) expression while maintaining cell viability (Fig. 1 B and Fig. S1 C). Next, we used these conditions to confirm the benefit of pooling multiple cr/tracrRNAs toward enhancement of target gene disruption. For example, eGFP-targeting crRNA sequences "cr1" and "cr3" were delivered as a pool for the monocyte eGFP screen. Shown in Fig. 1 C, individual crRNA sequences targeting eGFP conferred varying degrees of gene loss; however, pooling cr3 with either cr1 or cr2 provided maximal eGFP loss. Combining all three cr/tracrRNAs did not improve eGFP loss beyond the most effective pairings. Thus, we concluded that pooling two distinct cr/tracrRNAs targeting the same gene provides optimal gene KO in murine BMDMs or monocyte-derived macrophages.

DCs play a critical role in instructing T cell responses through the process of antigen presentation (reviewed in Merad et al., 2013). Therefore, we sought to extend our protocol for genetic modification of DCs. BM-derived DCs (BMDCs) serve as a model for investigating fundamental mechanisms of DC biology, and more broadly, engineered DCs may constitute the critical material for future cell-based vaccines. A traditional method of differentiating BM cells into DCs involves supplementation with GM-CSF (Cornel et al., 2018; Gundry et al., 2016). However, this does not produce the different physiologically relevant subsets found in vivo. We therefore decided to make use of a protocol that uses Flt3 ligand (Flt3l) supplementation to differentiate BM cells into at least three DC cell types found in vivo: two conventional DC (cDC) cell types (Sirpα^+^ DC and CD24^+^ DC), and plasmacytoid DC (pDC; B220^+^) cells (Naik et al., 2005). In addition, this differentiation method allows the production of a large number of BMDCs, which are otherwise rare in vivo. Both plasmid transfection and viral transduction methods have been developed for primary DC gene delivery, but aside from variable and inefficient delivery across the target cell population, these methods require the use of biohazardous material and/or may introduce potential cell toxicity or differentiation artifacts (Bowles et al., 2011).

**Figure S2.**
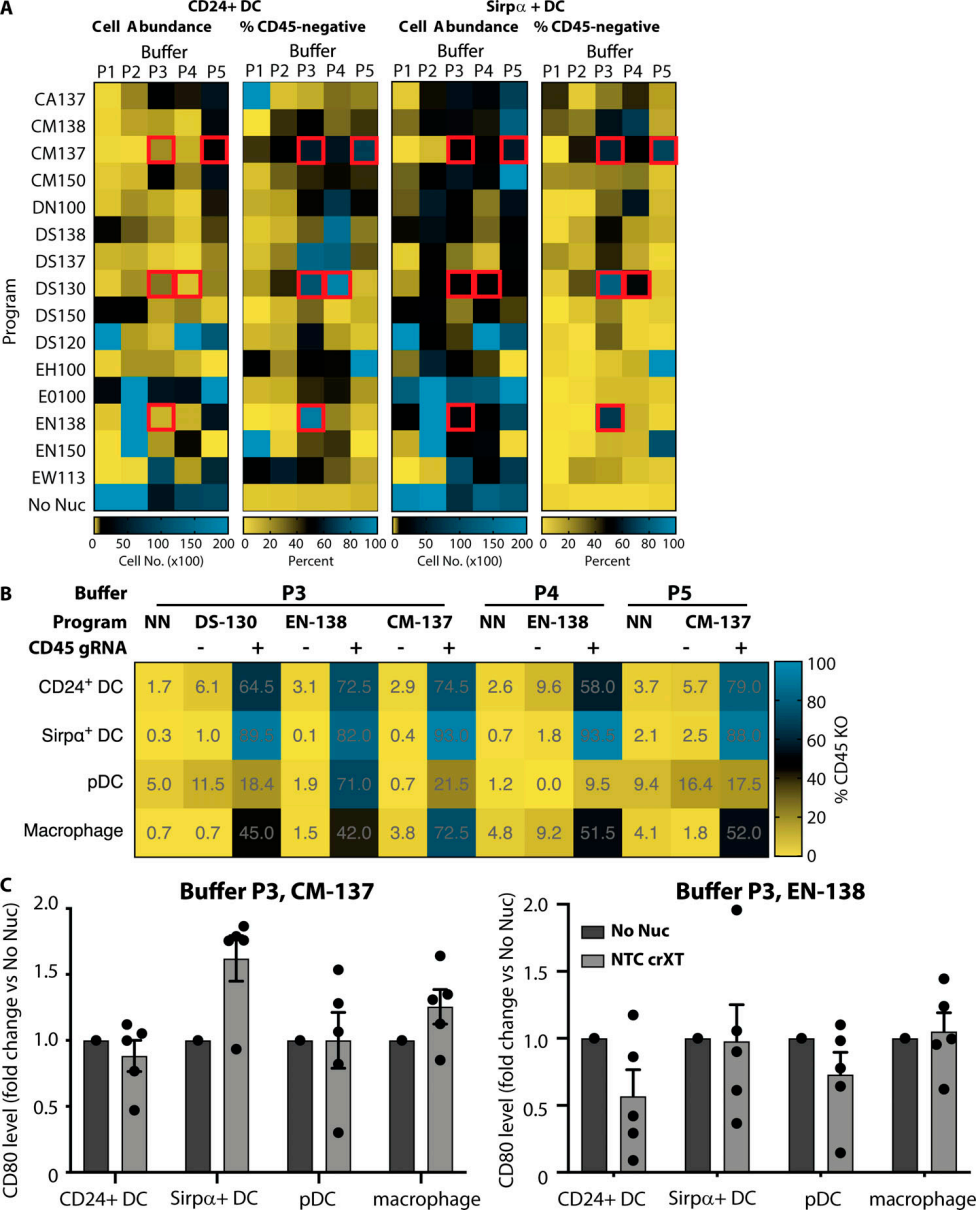
**Screening of optimal Cas9-RNP nucleofection protocols for KO in murine BMDCs. (A)** Data from the initial optimization screen are shown in four heatmaps reporting the cell abundances and CD45 KO efficiency in CD24^+^ (left panels) and Sirpα^+^ (right panels) DCs. Red boxes indicate the five conditions that showed the highest KO efficiency while maintaining acceptable cell abundance. Data are from one experiment. **(B)** Confirmation of deletion efficiency of the top five conditions from the initial optimization using CD45 crRNAXT-Cas9-RNPs compared with NTC crRNAXT-Cas9-RNPs and no-nucleofection (NN) controls. Data are averaged from technical replicates. **(C)** Relative CD80 levels as measured by flow cytometry in BM-derived CD24^+^ DC, Sirpα^+^ DC, pDC, and macrophage cells not nucleofected (No Nuc) or nucleofected with NTC crRNAXT-Cas9-RNPs using either P3/CM-137 or P3/EN138 combinations. Data are presented as mean ± SEM (*n* = 5) and collected from two independent experiments.

To develop the protocol for DC gene editing, we first assessed delivery of the Cas9-RNPs. Mimicking the BMDM and monocyte workflows, we performed a primary cell optimization screen in murine BMDCs with some modifications (Fig. S2 A). We used an improved guide chemistry for the crRNA (crRNAXT; IDT) that became available over the course of this study. In the context of BMDCs, we found that higher density of BM cells improved viability after nucleofection. Therefore, nucleofection efficiency was evaluated at 2 million cells per condition. Additionally, a single *Ptprc *(hereafter CD45)-specific targeting sequence and a lower RNP concentration (30 pmol or 5 µg Cas9) were used to increase the dynamic range for measuring potential improvements in KO efficiency. After RNP nucleofection, cells were cultured for 12 d with Flt3 ligand. DC and macrophage subsets were phenotyped by flow cytometry, and cell surface CD45 expression was quantified by antibody stain (Fig. 1 D). As in the macrophage screen, we observed substantial variability in both KO efficiency and viability for both the CD24^+^ DC and Sirpα^+^ DC populations across the 80 conditions tested, with CD24^+^ DCs displaying lower cell viability across the majority of conditions compared with Sirpα^+^ DCs (Fig. S2 A).

We then selected five conditions that resulted in substantial KO and acceptable viability: Buffer P3 with programs CM-137, DS130, and EN-138; buffer P4 with program DS-130; and buffer P5 with program CM-137 (Fig. S2 A). These conditions were tested with the higher Cas9-RNP concentration used in the macrophage screen, and this revealed that the combination of buffer P3 with program CM-137 (P3, CM-137) gave the highest KO for CD24^+^ DCs (74.5% KO), Sirpα^+^ DCs (93% KO), and macrophages (72.5% KO; Fig. S2 B). However, this combination generated a relatively poor KO efficiency within pDCs (21.5% KO). Instead, we found that buffer P3 with program EN-138 (P3, EN-138) was the most effective for the pDC subset (71% KO). Importantly, although this condition was not as broadly efficacious as buffer P3 with program CM-137, it still resulted in >50% KO across all tested DC cell types. Consistent with these results, when tested in cells from a distinct murine donor, P3, CM-137 showed high KO efficiency (>80% KO) in CD24^+^ DCs, Sirpα^+^ DCs, and macrophages but not in pDCs, while P3, EN-138 resulted in higher KO efficiency in pDCs (>50% KO) but displayed slightly lower and more variable KO efficiency across the other three cell types (Fig. 1 E). We conclude that when using the crRNA or crRNAXT gRNA chemistry, P3, CM-137 is the best condition for achieving the highest efficiency KO in all APCs other than the pDC subtype in the Flt3 ligand BMDC culture.

Because it is critical to maintain a normal cell state as part of the gene editing process, we tested expression levels of CD80, a costimulatory protein and myeloid cell activation marker, on day 12 after nucleofection with our two selected conditions. We found that, with the exception of Sirpα^+^ DCs, the expression of CD80 was similar between nonnucleofection controls and cells that had been nucleofected using either condition, suggesting that delivery of Cas9-RNP at day 0 by these methods does not trigger broad activation of murine BM–derived myeloid populations at the time of analysis (Fig. S2 C).

### Population-level gene disruption in human monocyte-derived DCs and macrophages

Few methods are available for effective, non-viral gene modification of primary human macrophages and DCs, which restricts direct phenotypic comparison of genes shared across species, the study of genes unique to humans, or potentially, ex vivo editing of these cells for direct therapeutic benefit. Encouraged by our results for murine monocyte-derived macrophages and DCs, we reasoned that conditions for delivery of Cas9-RNPs may be consistent between species and cell types. In addition, we sought to expand our findings from murine cells by comparing updated Cas9-RNP technology platforms in the human context. This included Cas9 proteins (sourced from different vendors) and gRNA variants.

**Figure 2.**
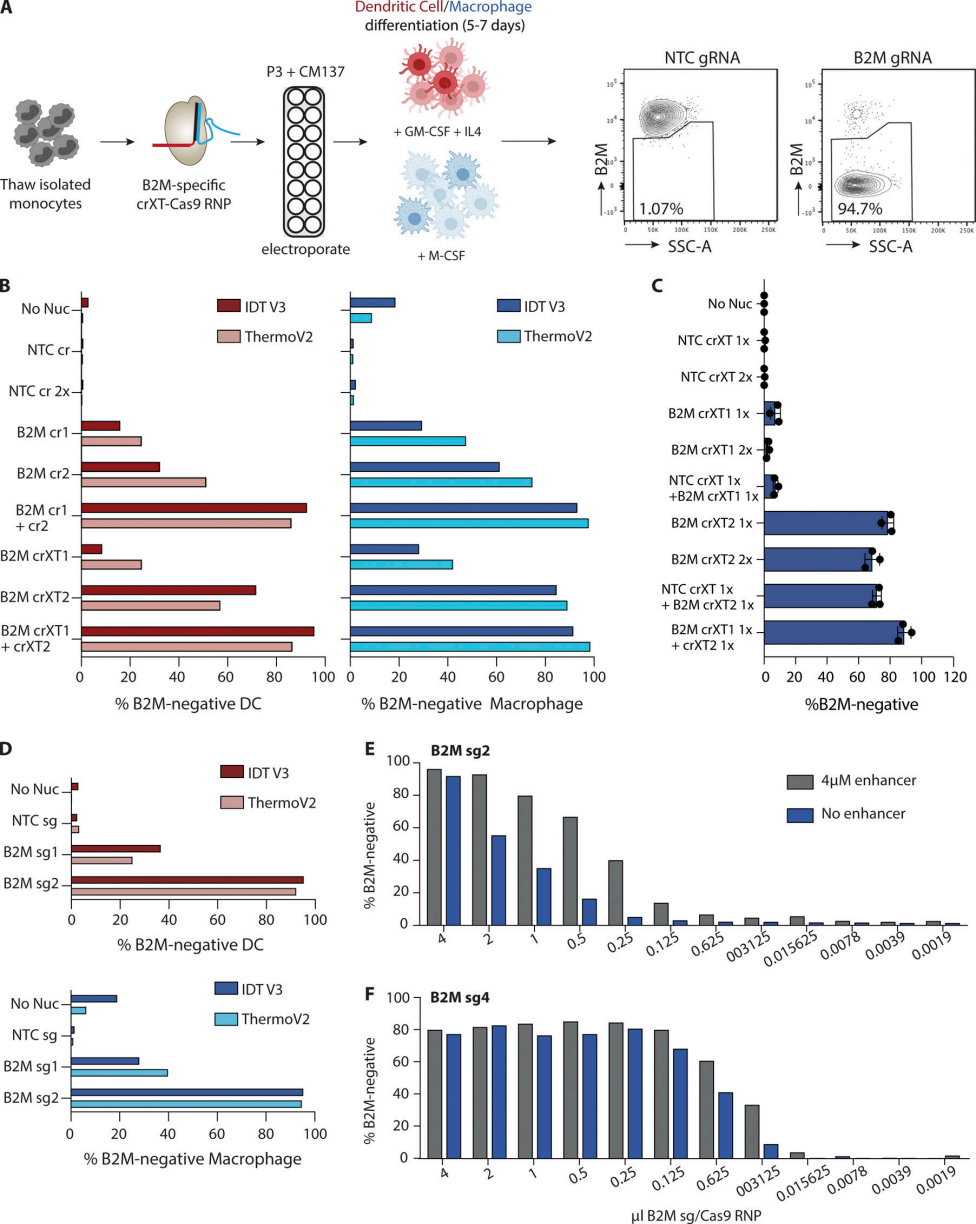
**Population-level gene editing in human monocyte-derived DCs and macrophages. (A)** Workflow for B2M-specific KO in human monocyte-derived dendritic and macrophage cultures. Representative flow cytometry plots show gating strategy for using Cas9-RNPs loaded with NTC gRNA to determine B2M-negative cells in each cell population. **(B)** B2M KO efficiency in monocyte-derived DC (pink, left bar graph) and macrophage (right, blue bars) cells nucleofected with distinct B2M targeting sequences in either crRNA (B2M cr1, B2M cr2) or crRNAXT (B2M crXT1, B2M crXT2) format, or NTC complexed with IDT V3 Cas9 (dark bars) or Thermo Fisher TruCut V2 Cas9 (light bars). Data are from one experiment. **(C)** B2M KO efficiency in monocyte-derived macrophages nucleofected with IDT V3 Cas9-RNPs loaded with two different crRNAXTs (crXT1 and crXT2) targeting B2M or NTC (NTC crXT). Cas9-RNPs were added individually or in combination. Each Cas9-RNP is labeled with 1× or 2× to indicate the relative molar quantity nucleofected into the cells. Data are presented as mean ± SD (*n* = 3). **(D)** Same as B, but gRNA-Cas9-RNPs were loaded with sgRNAs (B2M sg1 and sg2) instead of crRNAs or crRNAXTs. **(E and F)** Dose–response curve of B2M KO efficiency in monocyte-derived macrophages. IDT V3 Cas9-RNPs loaded with two different sgRNAs targeting B2M (sg2 or sg4) were nucleofected into cells at the indicated quantities. Cas9-RNPs were complexed and delivered with and without 4 µM of IDT electroporation enhancer. Data in B–D are representative of two independent experiments (*n* = 1 donor per experiment). Data in E and F are from one experiment (*n* = 1 donor). **(B–E)** No nucleofection (No Nuc) cells or cells nucleofected with NTC crRNA, crRNAXT, or sgRNA-Cas9-RNPs were used as controls. Buffer P3, CM-137 condition was used for all Cas9-RNP delivery.

To begin optimization of human myeloid cell editing, we targeted β2-microglobulin (encoded by the *B2m *gene, hereafter B2M), the broadly expressed constant region of the human MHC class I (MHCI) complex. This would permit assessment of gene deletion across multiple donors regardless of genetic background. We generated several RNP variants loaded with two distinct guide sequences (termed B2M cr/crXT1 and cr2) using standard (crRNA) or XT (crRNAXT) guide chemistry, alone or combined in each reaction, comprising either the V3 Cas9 protein from IDT or TruCut V2 Cas9 protein from Thermo Fisher Scientific. All RNPs were delivered into monocytes obtained from peripheral blood mononuclear cells (PBMCs) using buffer P3, CM-137; the generalizable condition identified in our murine assays (Fig. 2 A). After nucleofection of the B2M-specific Cas9-RNP variants, monocytes were differentiated into either macrophages (growth medium supplemented with M-CSF, ∼5 d culture) or DCs (growth medium supplemented with GM-CSF and IL-4, ∼7 d culture). The unique B2M targeting sequences exhibited different KO efficiencies, regardless of guide chemistry, with cr2 cutting more efficiently than cr1, and KO was generally higher in the macrophage subset. While the individual guide efficacy neared 90% population-wide KO, similar to our experiments performed with murine cells, pooling of Cas9-RNPs was able to induce near-complete KO (>90%) in both cell types (Fig. 2 B). As with the individual guides, the standard and XT gRNA pools produced similar results in these assays. Negligible differences were observed when comparing the Cas9 protein variant performance across all tested conditions.

### Cas9-RNPs show additivity in human myeloid cells

**Figure S3.**
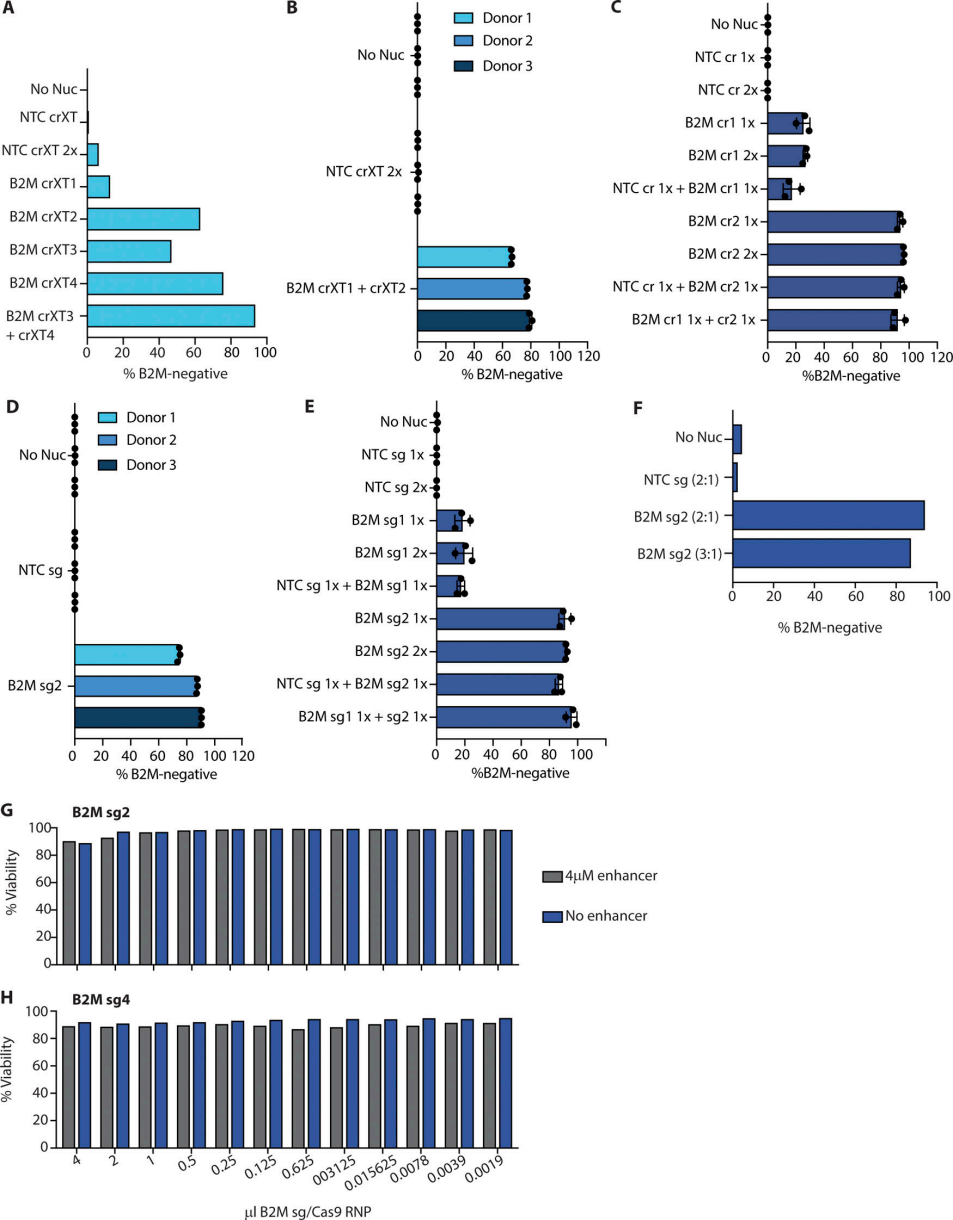
**Supporting data for population-level gene disruption in human monocyte-derived DCs and macrophages. (A)** B2M KO efficiency in monocyte-derived macrophages nucleofected with four different crRNAXTs (crXT1, crXT2, crXT3, and crXT4) targeted against B2M and complexed with Cas9. Data are from one experiment. **(B)** B2M KO efficiency in monocyte-derived macrophages nucleofected with two unique crRNAXTs (crXT1 and crXT2) targeted against B2M and complexed with Cas9. Data from three different donors are displayed, each performed in triplicate (*n* = 3). **(C)** B2M KO efficiency in monocyte-derived macrophages nucleofected with indicated crRNAs targeted against B2M or NTC and complexed with Cas9. **(D)** B2M KO efficiency in monocyte-derived macrophages nucleofected with a single sgRNA (sg2) and complexed with Cas9. Data from three different donors are displayed, each performed in triplicate (*n* = 3), and are displayed as mean ± SD. **(E)** B2M KO efficiency in monocyte-derived macrophages nucleofected with IDT V3 containing Cas9-RNPs loaded with two different sgRNAs (sg1 and sg2) targeting B2M or a NTC sgRNA (NTC sg). Cas9-RNPs were added individually or in combination. Each Cas9-RNP is labeled with 1× or 2× to indicate the relative molar quantity nucleofected into the cells. Data in C and E are mean ± SD (*n* = 3) and representative of two independent experiments. **(F)** B2M KO efficiency in monocyte-derived macrophages for IDT V3 Cas9-RNPs loaded with NTC sgRNA or B2M sgRNA2 and complexed with an sgRNA:Cas9 molar ratio of 2:1 or 3:1. **(G and H)** Cell viability in monocyte-derived macrophages from samples in Fig. 2, E and F. Data in F–H are from single donors (*n* = 1) and one individual experiment.

Across all mouse or human myeloid cell types we had tested through our initial optimization process, we routinely observed an apparent additive effect on gene disruption by combining multiple, unique gRNAs against the same target, regardless of guide chemistry. For example, this was observed in human cells for B2M-specific gRNA sequences 1 and 2, as well as a second set of B2M guides (sequences 3 and 4; Fig. S3 A). This effect was independent of the human donor (Fig. S3 B). Therefore, we decided to study this relationship in more detail.

Per the protocol used in our initial optimization scheme, gRNA pools were created by mixing equal parts of RNPs loaded with the unique guides before nucleofection, effectively generating a 2× stock compared with the individual guide preparations. While multiple targeting events may increase the probability of a frameshifting indel or a local chromosomal rearrangement, improving KO efficiency, it remains possible that the additive effect we observed with multiple guides was due to an elevated Cas9-RNP load introduced during the nucleofection step. To test whether RNP load was the determining factor, we compared delivery of 1× and 2× amounts of RNP with individual XT guides targeting B2M in human macrophages. As shown in Fig. 2 C, no appreciable difference in KO efficiency was observed for either gRNA 1 or 2, suggesting that the Cas9 protein amount was not limiting in the 1× condition.

Separately, we sought to evaluate whether the gRNAs act independently or cooperatively when pooled. Here, we mixed equal parts of either B2M-specific guide-Cas9-RNP with a non-targeting control guide (NTC)-Cas9-RNP. Addition of NTC gRNAs did not impact efficiency of gene editing in any crRNA format (Fig. 2 C and Fig. S3 C). These results demonstrate that total RNP concentration is not a significant limiting factor in these assays, and that comparative assessment of individual gRNAs can reveal highly potent candidates that function efficiently in isolation.

### Chemically synthesized single guide RNAs (sgRNAs) produce optimal gene disruption in primary myeloid cells

Two-part gRNAs provide a cost-effective and easy-to-produce solution for gene editing purposes. More recently, fully synthetic sgRNAs have been developed; these link both the crRNA and tracrRNA into a single unit (Jinek et al., 2012). Synthetic sgRNAs allow for chemical modification to increase function and/or stability and bypass the need for guide annealing before RNP complexing (Hendel et al., 2015; Kim et al., 2018; Ryan et al., 2018; Wienert et al., 2018). In accordance with their improved stability or function relative to two-part guides, we observed slight but overall enhanced KO efficiency in both monocyte-derived macrophages and DCs with B2M guides 1 and 2 formatted as sgRNAs (Fig. 2 D). This improvement was consistent across multiple donors (Fig. S3 D). Consistent with our findings using crRNA formats, we observed that Cas9 protein amount did not influence efficiency of gene KO, nor did the presence of an NTC sgRNA when combined with B2M-specific sgRNAs (Fig. S3 E). Pooling two sgRNAs targeting the same gene modestly improved KO efficiency, as observed with crRNA formats (Fig. S3 E).

We reasoned that the increased activity of sgRNAs might allow them to be used at a lower gRNA:Cas9 ratio compared with the 3:1 ratio we found to be optimal for crRNAs and crRNAXTs. In effect, this would aid in lowering costs associated with the use of synthetic sgRNAs. We therefore tested a 2:1 sgRNA:Cas9 ratio (B2M sgRNA2:Cas9) and found that this resulted in comparable KO efficiency relative to a 3:1 ratio (Fig. S3 F). Encouraged by the maintenance of activity with reduced gRNA concentration, we evaluated the minimum amount of sgRNA:Cas9 RNP required for effective target disruption. Here, we performed a titration experiment comparing a less active (sgRNA 2) versus a more active (sgRNA 4) sgRNA targeting B2M, with decreasing amounts of RNP in twofold increments. As shown for the less active guide (B2M sgRNA 2), KO efficiency dropped by 36.5% when the RNP volume was halved from 4 µl (180 pmol gRNA, 60 pmol Cas9) to 2 µl (90 pmol gRNA, 30 pmol Cas9; Fig. 2 E). We also performed the same titration curve in the presence of “electroporation enhancer” (a single-stranded DNA carrier, IDT; 4 µM) and found that, although this had no discernable effect on KO efficiency at 4 µl, the enhancer allowed for a reduction in the sgRNA2-RNP amount to 2 µl without substantial loss in KO efficiency (4 µl, 96.2%; 2 µl, 92.9%). Importantly, the enhancer had no effect on cell viability (Fig. S3, G and H). To our surprise, the activity curve for the more effective sgRNA displayed a significant shift, with the RNPs retaining near-complete activity at the 0.125 µl dose in the presence of the enhancer, or ∼16-fold less protein versus that needed for equivalent efficiency with the less-potent gRNA (Fig. 2 F). Taken together, these data show that a 2:1 sgRNA:Cas9 molar ratio in the presence of 4 µM enhancer is optimal, but the minimal amount of RNP to achieve maximal KO efficiency should be tested for each guide, as substantial reductions in the effective RNP quantities may be achievable.

**Figure S4.**
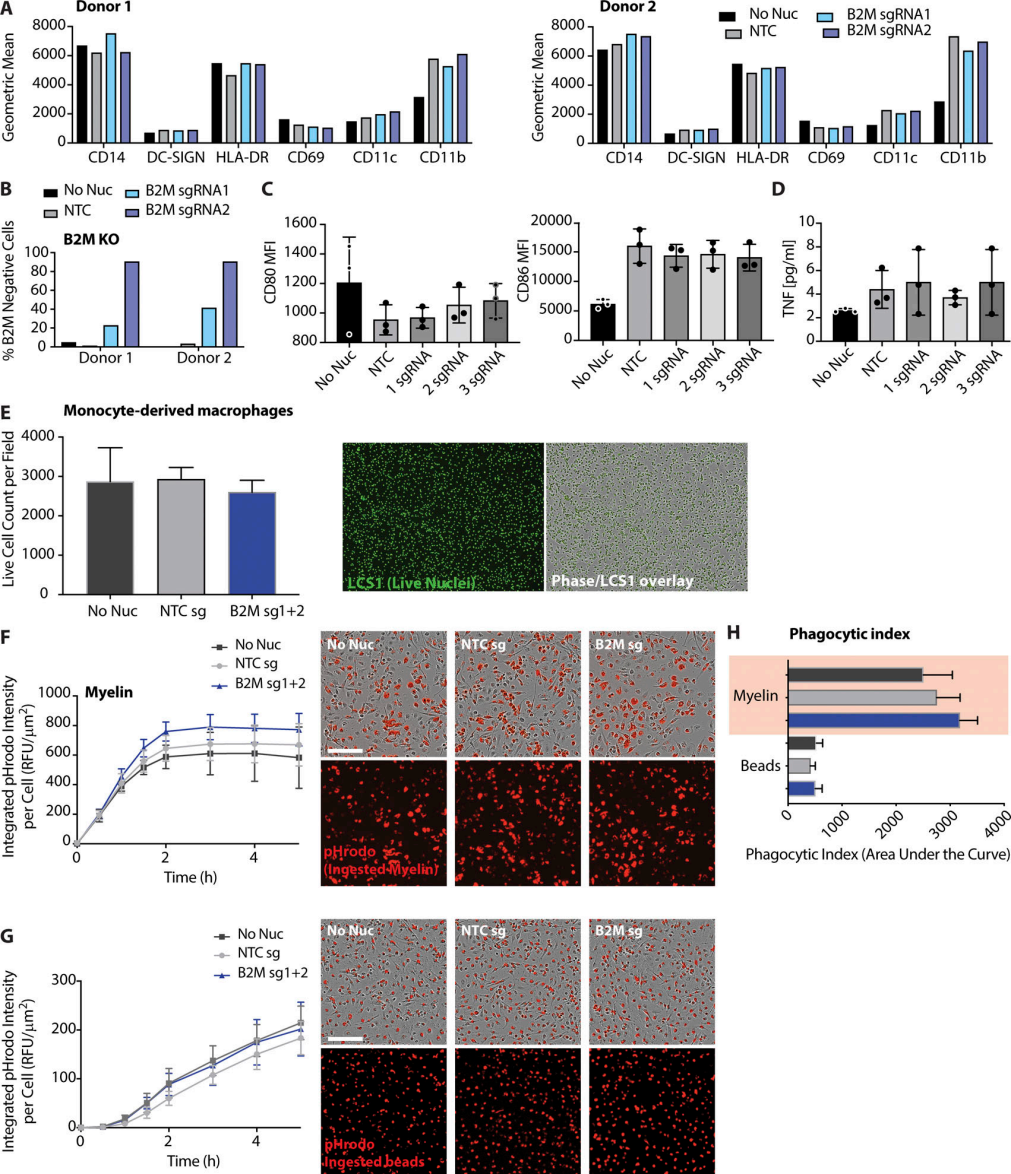
**Analysis of activation markers, cytokine release, and phagocytosis in human monocyte-derived macrophages following RNP nucleofection. (A)** Cell surface levels of indicated phenotypic markers measured by flow cytometry 5 d after nucleofection. **(B)** Efficiency of B2M-KO following nucleofection with two unique sgRNA:Cas9 RNPs. **(C)** Comparison of cell surface levels of CD80 and CD86 measured by flow cytometry 5 d after nucleofection. **(D)** ELISA measurements of TNF levels in cell culture medium of monocyte-derived macrophages following nucleofection. **(E)** Quantification of live monocyte-derived macrophages using imaging of live cell nuclei. Micrographs depict representative images of cultured cells. **(F and G)** Quantification of kinetics of particulate phagocytosis (F, myelin-pHrodo; G, beads-pHrodo). Micrographs depict representative images of phagocytosis following nucleofection, taken at the 5 h time point. Graphs in A–C depict MFI. Graphs in F and G depict intensity of pHrodo signal measured hourly following incubation with depicted particulate cargo. **(H)** Quantification of phagocytic index measured as AUC of data in G and H over 5 h of imaging. Data in A and B represent single measurements from two individual donors. Data in C–H are mean ± SD (*n* = 3) from three individual donors and a single experiment. Scale bars = 150 µm.

We next assessed the impact of sgRNA:Cas9 RNP nucleofection on innate cell activation and cytokine production. Measuring myeloid cell phenotypic markers on monocyte-derived macrophages from two independent donors revealed comparable cell surface levels of CD14, DC-SIGN, HLA-DR, CD69, and CD11c across all experimental conditions (Fig. S4 A). CD11b expression was elevated following nucleofection (Fig. S4 A). Importantly, the efficiency of gene deletion was not correlated with expression level of any of the tested phenotypic markers (Fig. S4 B). We also compared the impact of single or pooled sgRNAs on expression of costimulatory proteins and cytokines by monocyte-derived macrophages. Elevated levels of the costimulatory protein CD86 but not CD80 were observed upon nucleofection; these were independent of the quantity of gRNA (Fig. S4 C). Levels of secreted TNF remained low following nucleofection (Fig. S4 D), while IFNβ levels were undetectable in all conditions (not shown).

Finally, we characterized the impact of nucleofection on phagocytic capacity of monocyte-derived macrophages using live-cell imaging. Macrophages were coincubated with particulate cargo of varying size (myelin debris) or a defined diameter (beads) labeled with a pH-sensitive fluorescent dye (pHrodo-red) and imaged periodically over 5 h. This permits quantification of cargo uptake as well as delivery to the lysosomal (degradative) compartment of macrophages. Given that cell density has a significant impact on these measurements, live cell counts were determined immediately after nucleofection to ensure equal cell numbers in each experimental condition. Live cell counts at the end of the assay confirmed equivalent cell densities among nonnucleofected, nontargeted control (NTC sg) and B2M-KO (B2M sg) monocyte-derived macrophages (Fig. S4 E). We observed equal rates of phagocytosis of myelin (Fig. S4 F) as well as beads (Fig. S4 G). We generated a phagocytic index from the time-course data (area under the curve [AUC] over 5 h), which indicated comparable levels of phagocytosis by monocyte-derived macrophages in each treatment group (Fig. S4 H). We conclude that nucleofection results in a measurable difference for specific phenotypic and activation markers, but does not broadly induce a phenotypic change in monocyte-derived macrophages. These observations also provide impetus for researchers to use nonnucleofected as well as nontargeting gRNAs as standardized controls in their experimental design.

### Efficient CRISPR-Cas9 deletion of *Tlr7* in murine BMDCs

**Figure 3.**
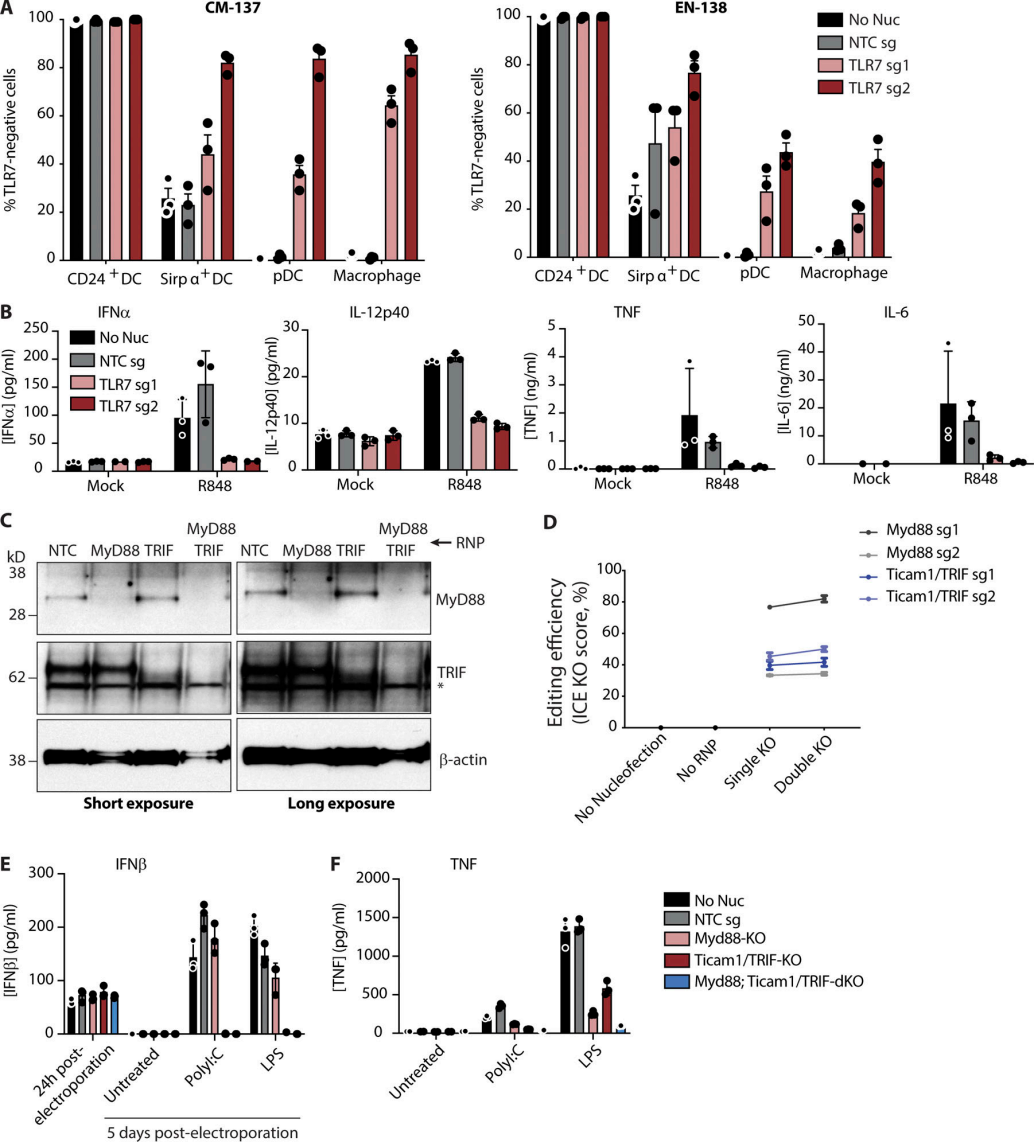
**Disruption of single or multiple genes in murine BMDCs and BMDMs to study TLR signaling. (A)** Percentage of cells that were TLR7 negative from BM-derived CD24^+^, Sirpα^+^ DCs, pDCs, and macrophages nucleofected with Cas9-RNPs loaded with an NTC or two different *Tlr7*-specific sgRNAs (sg1 and sg2). Left: Buffer P3, Program CM-137; Right: Buffer P3, Program EN-138. TLR7-negative cells were assayed by intracellular flow cytometry. Data are presented as mean ± SEM (*n* = 3, biological triplicates) and representative of two independent experiments. **(B)** Cytokine levels measured by Luminex in supernatant from BMDC culture (combined cell types) in A after stimulation with mock or 800 ng/ml of the TLR7 agonist R848 for 17 h. Data are presented as mean ± SD (*n* = 3, technical triplicates) and are representative of two independent experiments. **(C)** Representative Western blots depicting MyD88 or TRIF knockdown by sgRNA-Cas9-RNP in murine BMDMs. **(D)** Assessment of gene editing efficiency by Sanger sequencing 5 d after electroporation. Data are presented as mean ± SEM (*n* = 3) and representative of one experiment. **(E)** ELISA measurement of IFNβ levels in cell culture medium of BMDMs 24 h after electroporation and 5 d after electroporation treated as described. **(F)** ELISA measurements of TNF in cell culture supernatant following stimulation with the indicated ligand for 18 h. Data in E and F are presented as mean ± SD (*n* = 3) and representative of three independent experiments.

**Figure S5.**
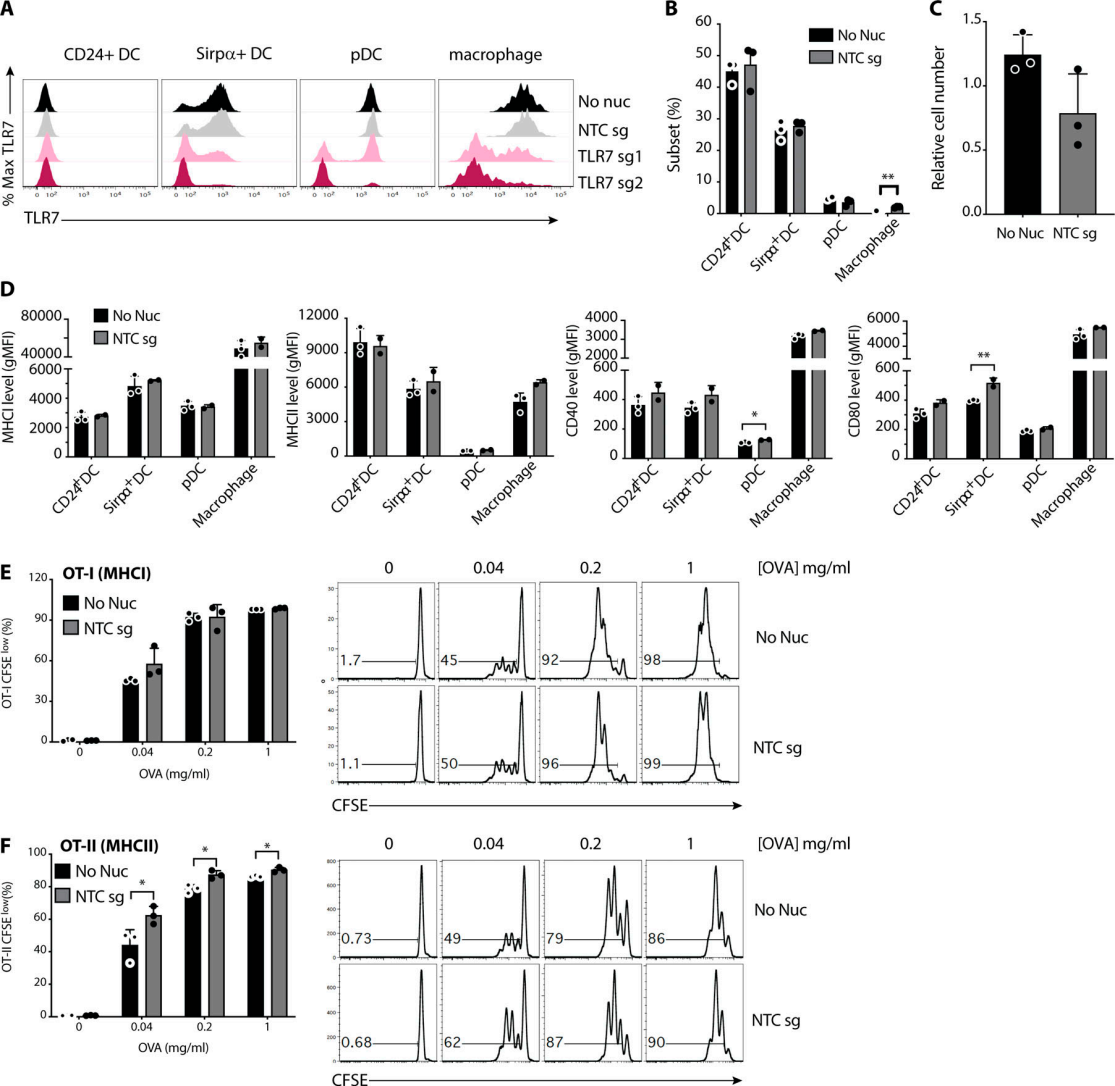
**Analysis of *Tlr7* editing efficiency and impact of nucleofection on BMDC phenotypes. (A)** Representative histograms of TLR7 flow cytometry following nucleofection with indicated RNP complexes using Buffer P3, Program CM-137. Quantification of TLR7-KO shown in Fig. 3 A. **(B)** Frequencies of indicated myeloid cell subsets 12 d following nucleofection and Flt3 ligand-mediated BMDC differentiation. **(C)** Relative abundance of BMDCs cultured in B. **(D)** Assessment of cell surface levels of indicated phenotypic and activation markers on BMDCs cultured as in B. **(E and F)** Quantification of CD8^+^ T cell/OT-I (E) or CD4^+^ T cell/OT-II (F) proliferation following 3 d of coculture with BMDCs nucleofected and pulsed with indicated concentrations of OVA. Histograms on right depict proliferation measured by CFSE dilution of OT-I or OT-II cells. Data in B–D are mean ± SEM (*n* = 3, biological triplicates) and are representative of two independent experiments. Data in E and F are mean ± SEM (*n* = 3, biological triplicates). *, P < 0.05; **, P < 0.01 derived using pairwise statistical analyses using an unpaired Student’s two-sided *t* test.

Having determined the optimal conditions for genetic manipulation of primary myeloid cell types, we next sought to determine whether these protocols could be used to study inflammatory responses. The general enhancements we observed with sgRNAs relative to crRNA variants prompted us to compare sgRNA gene-editing efficiency between the two optimal Cas9-RNP delivery conditions we had previously identified for Flt3 ligand–cultured BMDCs (buffer P3, program CM-137 vs. buffer P3, program EN-138 as in Fig. 1 E). We focused on TLR7, a microbe-associated pattern recognition receptor that is highly expressed on pDCs. Two *Tlr7*-specific sgRNAs of different efficiencies were nucleofected into mouse BM cells; these were differentiated for 12 d with Flt3 ligand. Using buffer P3, CM-137, we observed that individual sgRNAs led to efficient reduction of TLR7 in all myeloid cell subsets in a manner dependent on the inherent efficiency of each sgRNA (Fig. 3 A, CM-137; histograms in Fig. S5 A) with the exception of CD24^+^ DCs, which lacked detectable TLR7 even before nucleofection, as previously shown (Naik et al., 2005). In contrast, program EN-138 demonstrated decreased efficiency of *Tlr7* deletion in pDCs and macrophages (Fig. 3 A, EN-138). To quantify a cellular response to TLR7 activation, we determined the levels of multiple cytokines in the supernatant. *Tlr7* KO reduced levels of IFNα, IL-12p40, TNF, and IL-6 in the supernatant in response to stimulation with the TLR7 agonist R848, compared with the nonnucleofected and NTC sgRNA nucleofected samples (Fig. 3 B). This demonstrates that the protocol we have developed is able to generate efficient KO of *Tlr7* and abolish downstream cytokine responses in BMDCs while maintaining normal cell physiology.

To broadly assess the impact of sgRNA nucleofection on cell differentiation and function, we compared myeloid cell phenotypes, activation, and function following nucleofection with non-targeted sgRNA (NTC). While relative abundances of DC subsets were not impacted by nucleofection, we noted a modest (∼5%) increase in macrophage abundance compared with non-nucleofected cells (Fig. S5 B). A decrease in overall cellular yield was noted following nucleofection, likely an immediate consequence of nucleofection on the total BM at day 0 (Fig. S5 C). Comparing expression of activation and maturation markers revealed comparable levels of MHCI and MHCII across all cell subsets but elevated levels of CD40 on pDCs and CD80 on Sirpα^+^ DCs (Fig. S5 D). Finally, we compared MHCI- and MHCII-dependent antigen presentation. After nucleofection and differentiation as above, BMDCs were pulsed with varying concentrations of chicken OVA and cocultured with antigen specific CD8^+^ T cells (OT-I) or CD4^+^ T cells (OT-II). T cell proliferation was measured using flow cytometry after 3 d. OT-I proliferation was comparable between nonnucleofected and nucleofected conditions (Fig. S5 E). Nucleofection enhanced OT-II proliferation at all concentrations of OVA (Fig. S5 F). These changes, while mostly modest, require consideration when investigating specific myeloid cell subsets purified from the mixed culture system of Flt3 ligand–driven differentiation. Cumulatively, we recommend using buffer P3 and program CM-137 for optimal gene editing when using sgRNA chemistry.

### Multiplexed KOs of *Myd88* and *Ticam1*/TRIF in murine BMDMs

Next, we tested the ability of this protocol to generate single or multiple gene KOs in BMDMs to investigate TLR signaling. Macrophages use cytosolic adaptors MYD88 or TRIF (encoded by *Ticam1*) to engage TLRs for antimicrobial immunity. Whereas the bacterial cell wall antigen LPS activates TLR4 to signal via both adaptors, double-stranded RNA engages TLR3 and only signals via TRIF (Gay et al., 2014). Two sgRNAs per target gene were pooled to generate single or double KOs (Fig. 3 C). Sanger sequencing analysis of individual gRNAs revealed that *Myd88* sgRNA 1 exhibited the highest editing efficiency, whereas both *Ticam1*/TRIF sgRNAs exhibited ∼50% editing efficiency. These were unchanged in single or double KO conditions (Fig. 3 D; Sanger sequencing primers provided in Table S2). Measurement of IFNβ secretion by BMDMs was comparable across all conditions 24 h after nucleofection, demonstrating lack of an enhanced IFN response, and thus generalized activation, following Cas9-RNP delivery (Fig. 3 E). Engaging TLR3 with polyinosinic:polycytidylic acid (PolyI:C, a synthetic double-stranded RNA analogue) induced IFNβ and TNF by control (NTC) and *Myd88*-KO but not by those lacking *Ticam1*/TRIF (i.e., *Ticam1*/TRIF-KO or *Myd88*;*Ticam1*/TRIF-dKO; Fig. 3, E and F, PolyI:C treatment). Similarly, engaging TLR4 with LPS revealed that *Ticam1*/TRIF mutation was sufficient to abolish IFNβ secretion (Fig. 3 E, LPS treatment), whereas TNF secretion was dependent on a combination of MYD88 or TRIF signaling (Fig. 3 F, LPS treatment). Together, our results confirm the successful generation of compound KO BMDMs in a physiologically relevant context of innate antimicrobial immunity.

### Efficient multiplexing of gene KOs in primary human monocyte-derived macrophages

**Figure 4.**
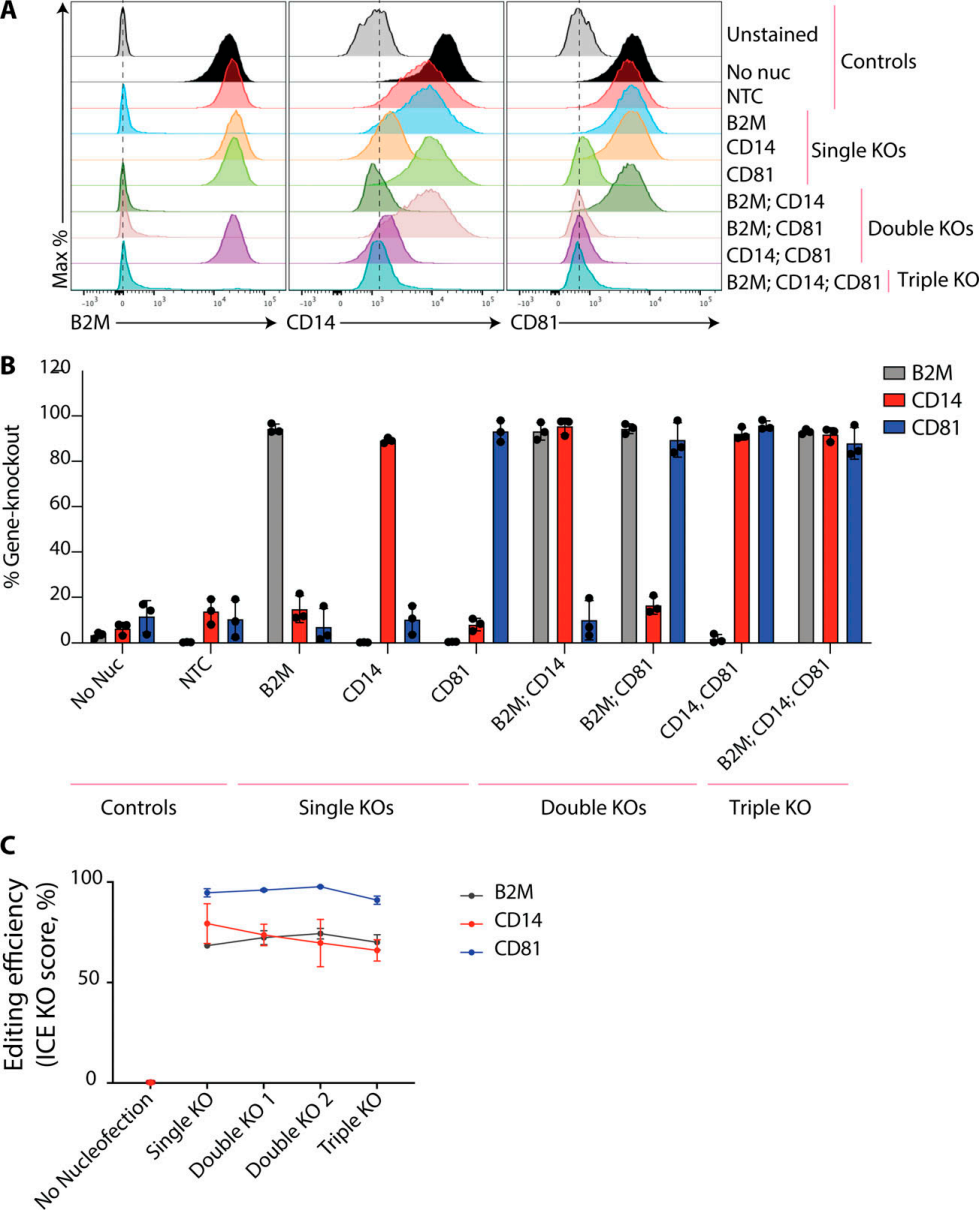
**Disruption of single and multiple genes in human monocyte-derived macrophages. (A)** Histograms depicting cell surface B2M, CD14, and CD81 protein levels in monocyte-derived macrophages as measured by flow cytometry. **(B)** Quantification of gene deletion as measured by flow cytometry. Data are presented as mean ± SD (*n* = 3) and representative of three independent donors. **(C)** Assessment of gene editing efficiency by Sanger sequencing 7 d after nucleofection. Data are mean ± SEM (*n* = 3) and representative of three independent donors.

We next sought to make single and multiplexed gene KOs in monocyte-derived macrophages from human donors. We chose to delete the genes encoding B2M along with CD14 and CD81, which would enable quantitative flow cytometry–based assessment of gene editing efficiency. A single sgRNA targeting each gene was introduced into monocytes by nucleofection using the P3, CM-137 protocol; cells were then differentiated into macrophages using M-CSF. On day 6 after nucleofection, cells were harvested to compare gene KOs. Flow cytometry revealed near-complete, population-wide editing in single, double, or triple KO samples (Fig. 4 A, representative histograms; Fig. 4 B, quantification of gene KO). Editing efficiency was confirmed by inference of CRISPR edits (ICE) analysis (Synthego), which provides a measure of mutant allele frequency across sequenced PCR products derived from the targeted loci (Fig. 4 C). Therefore, highly potent, individual gene-specific sgRNAs can be combined for one-step production of multiplex, population-wide KOs in primary human myeloid cell types without the need for selection or stable gene editing component expression.

## Discussion

We provide a rapid, efficient, and economical method to generate near-population-level gene KOs in primary myeloid cells of human and murine origins (summarized in Table 1 and Table 2). Optimizations developed herein permit single and multiplexed gene KOs (up to three genes at a time) with >90% efficiency, thus eliminating a need for stepwise gene disruption and/or transgenic marker selection. We reveal that combining pairs of gene-specific crRNAs provides an additive, optimal effect on KO efficiency. Alternatively, individual chemically synthesized sgRNAs can be mixed and used for near-complete, compound gene disruption. We also demonstrate that the addition of RNP electroporation enhancer in the context of human monocyte-derived cells can both considerably increase the gene editing efficiency of suboptimal gRNA sequences and reduce the effective dose of active sgRNAs. By increasing the repertoire of functional gRNA sequences while at the same time reducing the anticipated costs and material demands associated with generating a KO cell type, we have ameliorated separate barriers for high-throughput KO analysis in primary human and mouse myeloid cells. Given the observation that human monocytes up-regulated activation markers following nucleofection, it is prudent to monitor their activation states and use non-electroporated cells as controls when investigating innate inflammatory phenotypes. Comparison of additional markers revealed a lack of elevation following nucleofection, demonstrating that this protocol has limited, if any, effect on human myeloid cell activation status.

**Table 1.** Optimal conditions for Cas9-RNP–mediated gene deletion in murine myeloid cells

	Monocyte	Macrophage (M-CSF)	CD24^+^ DC (FLT3L)	Sirpα^+^ DC (FLT3L)	pDC (FLT3L)	Macrophage (FLT3L)
Program	CM-137	CM-137	CM-137	CM-137	EN-138 (crRNA, crRNAXT); CM-137 (sgRNA)	CM-137
Buffer	P3	P3	P3	P3	P3	P3
Cell number range per reaction in 20 µl volume	0.5–5 × 10^6^	0.5–5 × 10^6^	2–6 × 10^6^ total BM cells	2–6 × 10^6^ total BM cells	2–6 × 10^6^ total BM cells	2–6 × 10^6^ total BM cells
Culture conditions following electroporation	BMDM culture medium containing M-CSF	BMDM culture medium containing M-CSF	BMDC culture medium containing FLT3L	BMDC culture medium containing FLT3L	BMDC culture medium containing FLT3L	BMDC culture medium containing FLT3L

**Table 2.** Optimal conditions for Cas9-RNP–mediated gene deletion in human myeloid cells

	Monocyte-derived macrophage (M-CSF)	Monocyte-derived DC (GM-CSF + IL-4)
Program	CM-137	CM-137
Buffer	P3	P3
Cell number range per reaction in 20 µl volume	0.5–5 × 10^6^ fresh or thawed monocytes	0.5–5 × 10^6^ fresh or thawed monocytes
Culture conditions following electroporation	Monocyte-derived macrophage culture medium containing M-CSF	Mo-DC culture medium containing GM-CSF + IL-4

Relative to other cell types tested, we noted that gene editing efficiency in murine pDCs was markedly enhanced using sgRNAs versus crRNAs. Because pDCs predominantly differentiate from common lymphoid progenitors, a lineage distinct from myeloid lineage progenitors (Rodrigues et al., 2018), common lymphoid progenitors may be targeted more efficiently by sgRNAs. This observation warrants further assessment of CRISPR-Cas9-RNP–mediated gene editing in hematopoietic progenitors. It is also important to note that myeloid cells edited using our methods were cultured using varying cell adhesion conditions. For instance, murine monocyte-derived macrophages and BMDMs were maintained in low-attachment tissue culture (TC) multiwell plates or non–TC-treated Petri dishes, respectively. BMDCs were differentiated in conventional TC-treated multiwell plates. For assessment of gene KO and functional assays, BMDMs were initially cultured on Petri dishes and then transferred to TC-treated multiwell plates. Across all adhesion conditions, we observed comparable efficiency of gene KO. Thus, myeloid cell adhesion does not materially impact efficiency of gene editing or viability of cells following nucleofection with the optimized protocols generated herein.

We recently described the utility of this method in studying necroptosis, for which Cas9-RNP delivery into murine BMDMs was used to dissect signaling nodes of *Ticam1*/TRIF-mediated cell death (Lim et al., 2019). Beyond this example of reverse genetics, the scalability of our findings is likely to enable functional screening in primary myeloid cells. This is particularly relevant to human immunology given the known, but as yet broadly unexplored, phenotypic differences between human and murine immune cells. With our approach, arrayed platforms of focused gRNA libraries can be used to reveal phenotypes of interest in donor-derived human myeloid cells, followed by assessment in preclinical model systems such as mice. Furthermore, the ability to deliver significant quantities of recombinant Cas9 permits pooled functional screening in myeloid cells derived from KO or mutant mouse strains, as recently described for human lymphocytes (Ting et al., 2018). This alleviates the need to breed a genotype of interest with a Cas9 knock-in strain, thus providing significant advantages in study design and economy. Finally, the lack of significant chronic immune cell activation observed when using our methods generates the possibility of in vivo evaluation via adoptive cell therapy after RNP-mediated gene KO.

Our findings provide a significant technical advance in the study of myeloid cells, typically considered a challenging cell type for gene editing. Looking ahead, adapting our methods for alternative gene regulation (e.g., via CRISPRi or CRISPRa) or single-nucleotide polymorphism/reporter knock-in strategies will further expand our ability to investigate and modify innate immunity in the relevant primary cell subsets of interest.

## Materials and methods

### Mice

All mice in this study were used and all experiments were conducted following protocols approved by Genentech Institutional Animal Care and Use Committee. Female eGFP-transgenic mice were obtained from Jackson Laboratory (C57BL/6-Tg(CAG-EGFP)1Osb/J, 003291). Female wild-type C57BL/6J mice were obtained from Jackson Laboratory (000664). All mice were aged 8–12 wk.

### Human donors

Peripheral blood and PBMCs were collected from healthy donors participating in the Genentech blood donor program after written, informed consent from the Western Institutional Review Board.

### Human monocyte preparations

PBMCs were isolated using Sepmate tubes (StemCell Technologies) and ACK (ammonium-chloride-potassium) lysing buffer (Thermo Fisher Scientific) following the manufacturer’s protocol from buffy coats or leukopaks from healthy donors. Human monocytes were isolated from the PBMCs using the human monocyte isolation kit (Miltenyi) according to the manufacturer’s protocol. Monocytes were aliquoted and frozen in 10% DMSO and FBS for further use.

### Monocyte-derived DC and macrophage cultures

Monocyte-derived DCs were cultured in DC medium (RPMI supplemented with 10% FBS [Gibco], 2 mM l-alanyl-l-glutamine [GlutaMAX; Gibco], 55 µM β-mercaptoethanol [Gibco], 100 U/ml penicillin, 100 µg/ml streptomycin [Thermo Fisher Scientific], and cytokines GM-CSF 800 U/ml [Peprotech] and IL-4 500 U/ml [Peprotech]) at a density of 10^6^ cells/ml. Medium was changed every 2–3 d by removing half the volume of medium in the well, spinning down harvested cells at 400 ×*g* for 5 min, and resuspending spun cells in DC medium with 2× cytokines and replating with remaining cells. Monocyte-derived macrophages were cultured in macrophage medium (DMEM high glucose supplemented with 10% FBS [Gibco], 2 mM GlutaMAX, 100 U/ml penicillin, 100 µg/ml streptomycin, and M-CSF 100 ng/ml [Peprotech]). Medium was changed every 2–3 d by adding half the volume of medium with 1× cytokines into each well.

### Human monocyte-derived DC and macrophage KO

#### gRNA selection

All gRNA sequences were chosen using either the IDT predesigned guides (https://www.idtdna.com) or an in-house algorithm.

#### Preparation of cells

Cells were isolated as described above. Frozen monocytes were thawed into non–TC-treated 6-well plates (Thermo Fisher Scientific) at 10^6^ cells/ml in DC or macrophage medium with appropriate cytokines and cultured overnight.

#### Preparation of gRNAs

To prepare each gRNA, the Alt-R crRNA or crRNAXT, Alt-sgRNA, and Alt-tracrRNA (IDT) was reconstituted to 100 µM with Nuclease-Free Duplex Buffer (IDT). To prepare the crRNA/XT-tracrRNA duplexes, the two oligonucleotides were mixed at equimolar concentrations in a sterile PCR tube (e.g., 5 µl Alt-R crRNA, crRNAXT with 5 µl Alt-R tracrRNA). Oligos were annealed by heating at 95°C for 5 min followed directly by hybridization for 15 min at room temperature in a PCR thermocycler. The mix was then placed on ice or frozen at −20°C until further use.

#### Precomplexing of Cas9-RNPs

In either a sterile PCR strip or 1.5-ml tube, the annealed crRNA/XT-tracrRNA duplexes or sgRNAs were mixed with Cas9 (IDT Alt-R S.p. Cas9 Nuclease V3 or Thermo SpyCas9 TrueCut Cas9 Protein V2) at a 3:1 molar ratio for crRNA/XTs (3 µl of crRNA/XT-tracrRNA duplex + 2 µl Cas9 5 mg/ml) or for sgRNAs at a molar ratio of 2:1 (2 µl sgRNA + 2 µl Cas9 5 mg/ml) for each reaction, unless otherwise indicated. Cas9 protein and gRNAs were complexed by incubation at room temperature for ≥20 min.

#### Nucleofection of Cas9-RNPs

Appropriate medium (DC or macrophage) was prewarmed in a cell culture plate at 37°C for ≥30 min, and cells were harvested for nucleofection. For monocyte-derived DCs, the cells were collected from the 6-well plate and spun at 400 ×*g* for 5 min. Then ∼300 µl of 1× PBS was added to the each well, collected, and spun at 400 ×*g* for 5 min to harvest any remaining cells. The monocyte-derived macrophages were harvested similarly, with the exception that Detachin (Genlantis) was added after the PBS harvest, whereupon the 6-well plates were incubated for 5 min at 37°C to allow any attached cells to be released and collected. For both DCs and macrophages, the final cell pellets were then washed twice with ≥5 ml of 1× PBS and counted. 10^6^ cells per reaction were resuspended in 20 µl of P3 primary nucleofection solution (Lonza). The 20 µl cell/P3 nucleofection solution was then added to each Cas9-RNP complex and pipetted up and down three to five times gently to mix while avoiding bubbles. The cell/RNP mix was then immediately loaded into the supplied nucleofector cassette strip (Lonza). The strip was inserted into the Lonza 4D-Nucleofector (4D-Nucleofector Core Unit: Lonza, AAF-1002B; 4D-Nucleofector X Unit: AAF-1002X) and nucleofected with the Buffer P3, CM-137 condition. The cassette strip was removed, and 150–180 µl of prewarmed medium was immediately added into each cassette well. The medium/cell/RNP mix was pipetted into the appropriate cell culture dish, and the cells were cultured as described above for 5 d (macrophages) or 7 d (DCs). KO efficiency was subsequently assayed by flow cytometry or ICE.

#### Electroporation enhancer and Cas9-RNP titration analysis

Human monocyte-derived macrophage cells were harvested and electroporated with sgRNA-containing Cas9-RNPs using the P3, CM-137 condition as described above, with the following modifications. The amount of Cas9-RNP complex was titrated down from 4 µl (2 µl sgRNA + 2 µl Cas9 at 5 mg/ml) to 0.0019 µl with twofold dilutions. In the conditions including an electroporation enhancer, the same titration was performed with the addition of 1 µl (4 µM) of electroporation enhancer (IDT) to the Cas9-RNP complex before nucleofection during complexing.

### Flow cytometric analysis of human monocyte-derived DCs and macrophages

Cells were harvested as described above for nucleofection, then stained with LIVE/DEAD Fixable Aqua Dead Cell dye (Thermo Fisher Scientific) in 50 µl of 1× PBS for 10 min at room temperature. Cells were washed by the addition of 150 µl of 1× PBS to each well, and the cells were centrifuged for 3 min at 400 ×*g*. The pelleted cells were resuspended and incubated for ≥30 min with fluorophore-conjugated antibodies in 50 µl of FACS buffer at 4°C. Cells were washed as before and fixed with 2% PFA for 10 min at room temperature. Cells were then washed with 1× FACS buffer and resuspended in 180 µl of FACS buffer for analysis by flow cytometry. Monocyte-derived macrophages and DCs were identified by expression of CD64 or DC-SIGN, respectively. KO was determined by gating for negatively stained cells using the NTC. Heat-killed cells and unstained cells were used as controls for live-dead and positive antibody staining. All samples were analyzed by the FACSymphony system (BD).

### Cas9 editing validation by Sanger sequencing and ICE

#### Primer design

DNA primers were generated up to 350 bp up- and downstream of each locus being assessed. Genomic sequence was taken from NCBI RefSeq, and Primer3 was used for primer selection.

#### Genomic DNA (gDNA)

gDNA was extracted from cells using QuickExtract solution. Cells were washed with PBS, then 30 µl (96-well plate) or 100 µl (24-well plate) of QuickExtract solution was added to each well and incubated for 1–5 min at room temperature. The solution was pipetted to ensure recovery of cells and transferred to PCR tubes. Samples were vortexed briefly and incubated at 65°C for 6 min and then 98°C for 2 min. gDNA extracts were stored at −20°C until ready for use as PCR templates.

#### PCR

PCR was run with Terra polymerase (35 cycles) following the manufacturer instructions. 1 µl of gDNA was used per 20 µl reaction, and the annealing temperature was 68°C for all samples. One test reaction (edited gDNA) and one control reaction (unedited gDNA) was run for each sgRNA target editing site. Samples were run on an agarose gel to confirm a product of the appropriate size before purification with AMPure XP SPRI beads following manufacturer instructions. AMPure XP beads were added in a 1:1 (vol/vol) ratio, and the final elution was in 12 µl of water.

#### Sequencing and analysis

Clean PCR product was submitted for Sanger sequencing (Elim Biopharm) using the forward primer (∼250 bp upstream of the target editing site). Sanger results were aligned and analyzed using the ICE web application (https://ice.synthego.com).

### Phagocytosis assays

#### Substrate preparation

Phagocytosis substrates (beads and myelin debris) were labeled with the pH-sensitive fluorescent dye pHrodo. pHrodo Red succinimidyl ester (Thermo Fisher Scientific) was dissolved in DMSO to 10 mM to make a stock solution and stored at −20°C. 1.18-µm-diameter amino-functionalized polystyrene beads (Spherotech AP-10-10) were washed three times in PBS by subsequent rounds of centrifugation (14,000 ×*g* for 1 min) and resuspension, all at room temperature. After washes, beads were pelleted and resuspended to 2.5% wt/vol in PBS. pHrodo Red succinimidyl ester was added at 1 µl stock solution per 500 µl of 2.5% wt/vol beads. The solution was rapidly mixed, then protected from light and incubated on a nutator for 30 min at room temperature. Beads were then washed three times in PBS as before and resuspended in PBS to a final concentration of 2.5% wt/vol. Labeled beads were stored at 4°C protected from light. Myelin debris was prepared according to published protocols (Larocca and Norton, 2007). Briefly, 4–6 wk-old male Sprague-Dawley rats were euthanized by CO_2_ asphyxiation and perfused transcardially with ice-cold PBS to remove blood. Brains and purified myelin were kept on ice or at 4°C for all subsequent steps unless noted otherwise. Whole brains were isolated, minced, and homogenized using a Dounce homogenizer in a buffered solution containing protease inhibitors and 0.3 M sucrose. The homogenate was layered over a 0.83-M sucrose solution and subjected to ultracentrifugation (75,000 ×*g* for 30 min). The interphase layer was collected, subjected to homogenization again, and diluted into a hypotonic buffer solution to rupture any remaining cells and organelles. Myelin debris was then washed twice by centrifugation (6,000 ×*g* for 15 min) and resuspension in the hypotonic solution. After two washes, myelin debris was resuspended in 0.3-M sucrose solution, layered over 0.83-M sucrose, and again subjected to ultracentrifugation as before. The interphase was collected and washed twice in hypotonic solution to yield purified myelin debris. The final pellet was weighed for use in subsequent concentration calculations. The purified myelin debris was transferred to Eppendorf tubes (100 mg per tube in 1 ml) and washed three more times in PBS (centrifugation at 14,000 ×*g* for 3 min) before resuspension in PBS to 50 mg/ml. pHrodo Red succinimidyl ester was added at 0.3 µl stock solution per 1 ml of 50 mg/ml myelin. The solution was rapidly mixed and then protected from light and incubated on a nutator for 30 min at room temperature. Myelin was then washed three times in PBS as before and resuspended in PBS to a final concentration of 50 mg/ml. Myelin was distributed into 125 µl aliquots and stored at −20°C until use.

#### Cell preparation and assay

Banked human monocytes were thawed, and nucleofection was performed as described above for both monocyte-derived macrophages and monocyte-derived DCs. Live cells were counted a second time using a Cellometer K2 (Select Science, 207101) immediately after nucleofection to plate cells from the nonnucleofection condition and the two nucleofection conditions at the same starting density. This was necessary to account for cell loss during nucleofection. Cells were diluted to 10^6^ cells/ml, and 100 µl was added to each well of 96-well TC plates (Costar, 3595). Cells were cultured with regular media changes as described above. On day 5 after nucleofection, pHrodo-labeled beads or pHrodo-labeled myelin debris were diluted into the appropriate growth medium, 100 µl of 50 mg/ml myelin debris suspension per 5 ml of medium or 25 µl of 2.5% wt/vol bead suspension per 5 ml of medium, and 50 µl of the diluted suspension was added to each well. Cells were quickly returned to the incubator and imaged at 30–60-min intervals on red and phase channels using an Incucyte S3 live cell analysis system. Reported values are averaged over four fields per well (10× objective), with at least eight wells used per data point. After the last imaging time point (5 h), the live nucleus stain Nuclear Green LCS1 (Abcam, ab138904) was added to a final concentration of 6 µM, and cells were returned to the incubator. 15 min after addition of LCS1 dye, a final image was acquired on the green channel to determine the total number of live cells per field. To calculate a phagocytic index for each well, the total pHrodo-positive area was determined for each time point and normalized to the total LCS1 live cell count measured immediately after the 5 h time point. Total pHrodo-positive area or live nuclei counts were calculated using automated analysis scripts that performed a fixed-value adaptive background subtraction and selected signal-positive regions that passed intensity thresholds. Accurate selection of pHrodo or calcein signal was confirmed visually. The integrated pHrodo intensity per cell was plotted over time, and AUC was used for statistical comparisons between treatments.

### Murine BMDM culture

BM was harvested from tibias and femurs of mice. Total BM cells were plated in BMDM culture medium (DMEM high glucose, 10% FBS [VWR], GlutaMAX, and penicillin/streptomycin supplemented with 50 ng/ml recombinant murine M-CSF [Genentech]) at a density of 0.5 × 10^6^ cells/ml in 150-mm non–TC-treated Petri dishes (VWR) in a volume of 20 ml per dish. After 2 d, 20 ml fresh BMDM culture medium was added without removal of medium. On day 4, all medium was removed, and 20 ml fresh BMDM culture medium was added. On day 5, cells were gently scraped from dishes using a rubber policeman and transferred to a 50-ml conical tube. The Petri dish was washed once with 20 ml of 1× PBS, and cells were harvested via centrifugation. Cells were resuspended in 10 ml of 1× PBS and counted, after which they were centrifuged once again for resuspension in appropriate buffers or media for downstream assays.

### Murine BMDM *Itgam*/CD11b CRISPR-Cas9 optimization screen

Day 5 BMDMs were resuspended in nucleofection solutions for primary cells (Primary Cell Optimization 96-well Nucleofector Kit, Lonza, V4SP-9096) at a density of 5 × 10^5^ cells per reaction in 20 µl nucleofection solution and mixed with Cas9-RNP containing B2M targeting gRNAs. This mixture was nucleofected using the Lonza 4D Nucleofector (4D-Nucleofector Core Unit: Lonza, AAF-1002B; 4D-Nucleofector X Unit: AAF-1002X). Immediately after nucleofection, ∼180 µl of prewarmed BMDM culture medium was added to each well, and cells were harvested by gently washing the well. Each reaction was transferred to a single well in a 6-well TC-treated plate containing 2 ml of prewarmed BMDM culture medium. Cells were cultured for an additional 5 d with complete medium changes at 2 and 4 d after nucleofection. On day 5, cells were harvested by gently scraping them with a rubber policeman. Harvested cells processed for flow cytometry. First, cells were stained with an Fc-Blocking reagent (CD16/32 FcR block, BD Biosciences) for 10 min at 4°C, followed by staining with an antibody cocktail for CD11b (anti-CD11b-APC) and CD45 (anti-CD45-FITC). Cells were washed twice in flow cytometry buffer and resuspended in flow cytometry buffer containing a viability marker (propidium iodide). Flow cytometry was performed to compare each nucleofection condition using a BD Fortessa analyzer. Loss of cell surface CD11b, shown as a drop in its MFI along with maintenance of cell viability, shown as a lack of propidium iodide–positive signal, was used to rank each condition.

### Murine monocyte culture and eGFP CRISPR-Cas9 optimization screen

BM was harvested from tibias and femurs of eGFP-transgenic mice. Red blood cells were lysed with ACK lysis buffer. Monocytes were isolated using a negative selection kit (Miltenyi). Cells were washed once with 1× PBS and resuspended in the nucleofection solutions for primary cells (Primary Cell Optimization 96-well Nucleofector Kit, Lonza) at a density of 2 × 10^5^ cells per well. Cells were nucleofected as above and immediately transferred to 6-well TC-treated plates containing prewarmed BMDM culture medium. Cells were cultured for 5 d, with complete medium changes at 2 and 4 d after nucleofection. On day 5, monocyte-derived macrophages were harvested by gently scraping them with a rubber policeman. Harvested cells were processed for flow cytometry. First, cells were stained with an Fc-Blocking reagent (CD16/32 FcR block, BD Biosciences) in the presence of a fixable viability dye (eFluor 780) for 20 min at 4°C in 1× PBS. Cells were washed twice in flow cytometry buffer, followed by staining for F4/80 (anti-F4/80-BV421). Cells were washed twice, and flow cytometry was performed to compare each nucleofection condition using a BD Fortessa analyzer. Loss of eGFP in the F4/80-positive population, along with maintenance of cell viability, shown as a lack of APC-Cy7–positive signal, was used to rank each condition.

### CRISPR-Cas9-mediated KO in murine BMDCs

BM cells were prepared, and red blood cells were lysed with ACK lysis buffer. BM cells were washed twice with 1× PBS and electroporated in the appropriate primary nucleofection solution (Primary Cell Optimization 4D-Nucleofector X Kit, P3 Primary Cell 4D-Nucleofector X Kit) using the Lonza 4D Nucleofector (4D-Nucleofector Core Unit: Lonza, AAF-1002B; 4D-Nucleofector X Unit: AAF-1002X) as described above. Specifically, 2 × 10^6^ BM cells per reaction were resuspended in 20 µl of primary nucleofection solution and mixed with Cas9-RNP containing targeting or NTC gRNAs. The cell + Cas9-RNP mix was then nucleofected with the appropriate program. Nucleofected cells were cultured in prewarmed RPMI medium supplemented with 10% FBS, l-glutamate, Hepes, antibiotics, β-mercaptoethanol, and 100 ng/ml Flt3 ligand (Peprotech) for 12 d in round-bottom 96-well TC-treated plates.

### Flow cytometric analysis of murine *Tlr7* KO BMDCs

Harvested BMDCs were stained with fixable viability dye (Thermo Fisher Scientific) in 1× PBS for 20 min. The centrifuged cell pellets were preincubated with FACS buffer containing CD16/32 Fc-block for 10 min. Cells were incubated for additional 30 min with fluorophore-conjugated antibodies in FACS buffer. Cells were washed and fixed with 2% PFA for 20 min before running flow cytometry. All staining was performed on ice. Subsets of cells in the BMDC cultures were gated as shown in Fig. 1 D. For intracellular staining of TLR7, a cell fixation and permeabilization kit was used as instructed (BD Biosciences). All samples were analyzed with the FACS Fortessa system (BD). Measurement of BMDC cytokine secretion was performed by Luminex assay.

### OT-I and OT-II proliferation following coculture with nucleofected BMDCs

BM cells were prepared, and red blood cells were lysed with ACK lysis buffer. The BM cells were washed twice with 1× PBS and nucleofected in P3 Primary Cell with either CM-137 program using 4D-Nucleofector X Kit (V4XP-3032) using the Lonza 4D Nucleofector (4D-Nucleofector Core Unit: Lonza, AAF-1002B; 4D-Nucleofector X Unit: AAF-1002X). Specifically, 2 × 10^6^ BM cells per reaction were resuspended in 20 µl of primary nucleofection solution and mixed or not with V3 Cas9-RNP complexed with nontargeting sgRNA. The cell + Cas9-RNP mix was then electroporated or not with either CM-137 program. Nucleofected cells were cultured in prewarmed RPMI medium supplemented with 10% FBS, l-glutamate, Hepes, antibiotics, 2-mercaptoethanol, and 100 ng/ml Flt3 ligand for 9 d in round-bottom 96-well TC-treated plates. On day 9, naive CD8 and CD4 T cells were isolated from splenocytes of OT-I and OT-II, respectively (EasySep Mouse Naive CD8+ T cell isolation kit and EasySep and Mouse Naive CD4^+^ T cell isolation kit, Stemcell Technologies), followed by carboxyfluorescein succinimidyl ester (CFSE) labeling (CellTrace CFSE Cell Proliferation Kit, ThermoFisher). KO BMDCs were preincubated with OVA protein of indicated concentrations for 1.5 h and washed twice with PBS. The OVA-pulsed BMDCs were cocultured with either naive CD8 T cells from OT-I or naive CD4 T cells from OT-II. After 3 d, the samples were analyzed by flow cytometry to measure the proliferation of T cells by CFSE dilution. For biological triplicates, the BM cells were prepared from three different B6 mice and were nucleofected, cocultured, and analyzed separately.

### TLR3 and TLR4 stimulation of gene-edited murine BMDMs

CRISPR-mediated KO was performed by nucleofecting day 5 wild-type BMDMs with nontargeting sgRNA or a pool of two sgRNAs targeting *Myd88* or TRIF (encoded by *Ticam1*) either in isolation or in combination. 5 × 10^6^ BMDMs were used per reaction (Buffer P3, program CM-137). Immediately after nucleofection, cells were transferred to 10-cm non–TC-treated Petri dishes containing 10 ml of prewarmed BMDM culture medium. Cells were cultured for an additional 5 d, with medium changes at day 2 and 4 after nucleofection. On day 5, cells were gently scraped and replated in TC-treated multiwell plates at a density of 0.5 × 10^6^ cells/ml for stimulation. BMDMs were stimulated overnight (18 h) with 100 ng/ml ultrapure LPS or 10 µg/ml PolyI:C low molecular weight (InvivoGen) to activate TLR4 or TLR3, respectively. Medium was collected after treatment for TNF (Invitrogen) and IFNβ (PBL Assay) measurements by ELISA. Gene disruption was assessed by Western blot.

### Western blot analysis

Assessment of CRISPR KO of murine BMDMs for deletion of *MyD88* and *Ticam1*/TRIF was performed by Western blotting. The cells were pelleted and washed twice with 1× PBS, and the pellet was resuspended in radioimmunoprecipitation assay buffer supplemented with protease inhibitors (Roche). Lysates were clarified by spinning for 10 min at 13,000 rpm at 4°C, and the protein content was measured by BCA (Pierce, 23225). SDS-PAGE was performed using a 4–12% gradient Bis-Tris gel (Novex) followed by protein transfer onto PVDF membranes and standard downstream immunoblotting. Chemiluminescence was detected by enhanced chemiluminescence (Western Lightning-plus ECL, PerkinElmer).

### Antibodies

Murine: I-A/I-E-BV421, B220-BV605, F4/80-BV711, Sirpα-PE-Cy7, CD45-FITC, TCR Vβ5.1, 5.2-APC, BV421-CD4, PE-Cy7 CD8a, PerCP-Cy5.5-CD11b, CD40-BV786, H2K^b^/H-2D^b^-Alexa647 (BioLegend); TLR7-PE, CD24-BUV395, CD80-BUV737, F4/80-BV421, CD11b-APC (BD); and eBioscience Fixable Viability Dye eFluor 780 (Thermo Fisher Scientific). Western blotting: TRIF (Genentech,), MYD88 (Abcam), β-actin (Cell Signaling Technologies). Human: CD64-APC, CD80-BV786 (BD); CD163-FITC, B2M-PE, CD209-APC (DC-SIGN); CD86-PE, CD80-PE-Cy7 (BioLegend); and CD14-PerCP-eFluor 710, CD81-FITC (Invitrogen). LIVE/DEAD Fixable Aqua Dead Cell dye and LIVE/DEAD Fixable Blue Dead Cell Stain Kit for UV excitation were from Thermo Fisher Scientific.

### Statistical analysis

Where depicted, pairwise statistical analyses were performed using an unpaired Student’s two-sided *t* test. Scatterplot and bar graphs reflect means of data. GraphPad Prism was used for data analysis and representation.

### Online supplemental material

Fig. S1 shows a screening of optimal Cas9-RNP nucleofection protocols for KO in murine monocytes and BMDMs. Fig. S2 shows a screening of optimal Cas9-RNP nucleofection protocols for KO in murine BMDCs. Fig. S3 shows supporting data for population-level gene disruption in human monocyte-derived DCs and macrophages. Fig. S4 shows an analysis of activation markers, cytokine release, and phagocytosis in human monocyte–derived macrophages following RNP nucleofection. Fig. S5 shows an analysis of *Tlr7* editing efficiency and impact of nucleofection on BMDC phenotypes. Table S1 lists gRNA sequences used in the study. Table S2 lists primer sequences for validation of gene editing by Sanger sequencing.

## Supplementary Material

Table S1 lists gRNA sequences.

Table S2 lists the primer sequences to validate Cas9 editing by Sanger sequencing.
