# Supplementary material for: Efficient gene knockout in primary human and murine myeloid cells by non-viral delivery of CRISPR-Cas9
Source: J Exp Med. 2020 May 1;217(7):e20191692. doi: 10.1084/jem.20191692 (PMC7336301; doi:10.1084/jem.20191692)
Supplement: Table S1 — lists gRNA sequences. [file JEM_20191692_TableS1.docx]

**Table S1. gRNA sequences**

| *Human* |  |
| --- | --- |
| B2M gRNA1 | AAGTCAACTTCAATGTCGGA |
| B2M gRNA2 | CGTGAGTAAACCTGAATCTT |
| B2M gRNA3 | ACTCACGCTGGATAGCCTCC |
| B2M gRNA4 | GAGTAGCGCGAGCACAGCTA |
| CD14 gRNA | GTAGATACAACTGACCCTGT |
| CD81 gRNA | GTTGGCTTCCTGGGCTGCTA |
| *Mouse* |  |
| CD11b/Itgam gRNA1 | TGCAGTACTCGGACGAGTTC |
| CD11b/Itgam gRNA2 | TTATAAGGATGTCATCCCCG |
| eGFP gRNA1 | GGTGGTGCAGATGAACTTCA |
| eGFP gRNA2 | GGAGCGCACCATCTTCTTCA |
| eGFP gRNA3 | GGCATCGACTTCAAGGAGGA |
| CD45 gRNA | AAACGCCTAAGCCTAGTTGT |
| TLR7 sg1 | TGTGCAGTCCACGATCACAT |
| TLR7 sg2 | ATCGAGGGCAATTTCCACTT |
| TRIF/Ticam1 sg1 | TCTGGTGTGTCAATGGGACG |
| TRIF/Ticam1 sg2 | CAAGCTATGTAACACACCGC |
| MYD88 sg1 | CCCACGTTAAGCGCGACCAA |
| MYD88 sg2 | GTCTGCGGGAGACCCCCGCG |
| *Control* |  |
| NTC gRNA | CGTTAATCGCGTATAATACG |
