# Supplementary material for: Efficient gene knockout in primary human and murine myeloid cells by non-viral delivery of CRISPR-Cas9
Source: J Exp Med. 2020 May 1;217(7):e20191692. doi: 10.1084/jem.20191692 (PMC7336301; doi:10.1084/jem.20191692)
Supplement: Table S2 — lists the primer sequences to validate Cas9 editing by Sanger sequencing. [file JEM_20191692_TableS2.docx]

**Table S2. Primer sequences to validate Cas9 editing by Sanger sequencing**

|  | Forward Primer | Reverse Primer | ICE score |
| --- | --- | --- | --- |
| mTicam1 sg1 | TCTGGTGTGTCAATGGGACG | CACTTGTGTCTGGAGCAGC | 51 |
| mTicam1 sg2 | AGAATGAGGCCTGGGATCG | TTTCTGTGGTGGACGTCAGG | 60 |
| mMyd88 sg1 | AGTCCATCCACCTTGATCGG | TCCTCACGGTCTAACAAGGC | 86 |
| mMyd88 sg2 | CCAGATCGCCTAGTCCATCC | ATCCAACAAACTGCGAGTGG | 37 |
| hB2M sg1 | TGAGAGGGCATCAGAAGTCC | AAGTCACATGGTTCACACGG | 88 |
| hCD14 sg1 | ACGATGAAGATTTCCGCTGC | CAGGTCTAGGCTGGTAAGGG | 72 |
| hCD81 sg1 | ACCCTACATCTTCCCAGCTG | TGCTCCCTGTTATATTTGTGGC | 95 |
